# Supplementary material for: Mining for Oxysterols in Cyp7b1−/− Mouse Brain and Plasma: Relevance to Spastic Paraplegia Type 5
Source: Biomolecules. 2019 Apr 13;9(4):149. doi: 10.3390/biom9040149 (PMC6523844; doi:10.3390/biom9040149)

S1

Figure S1

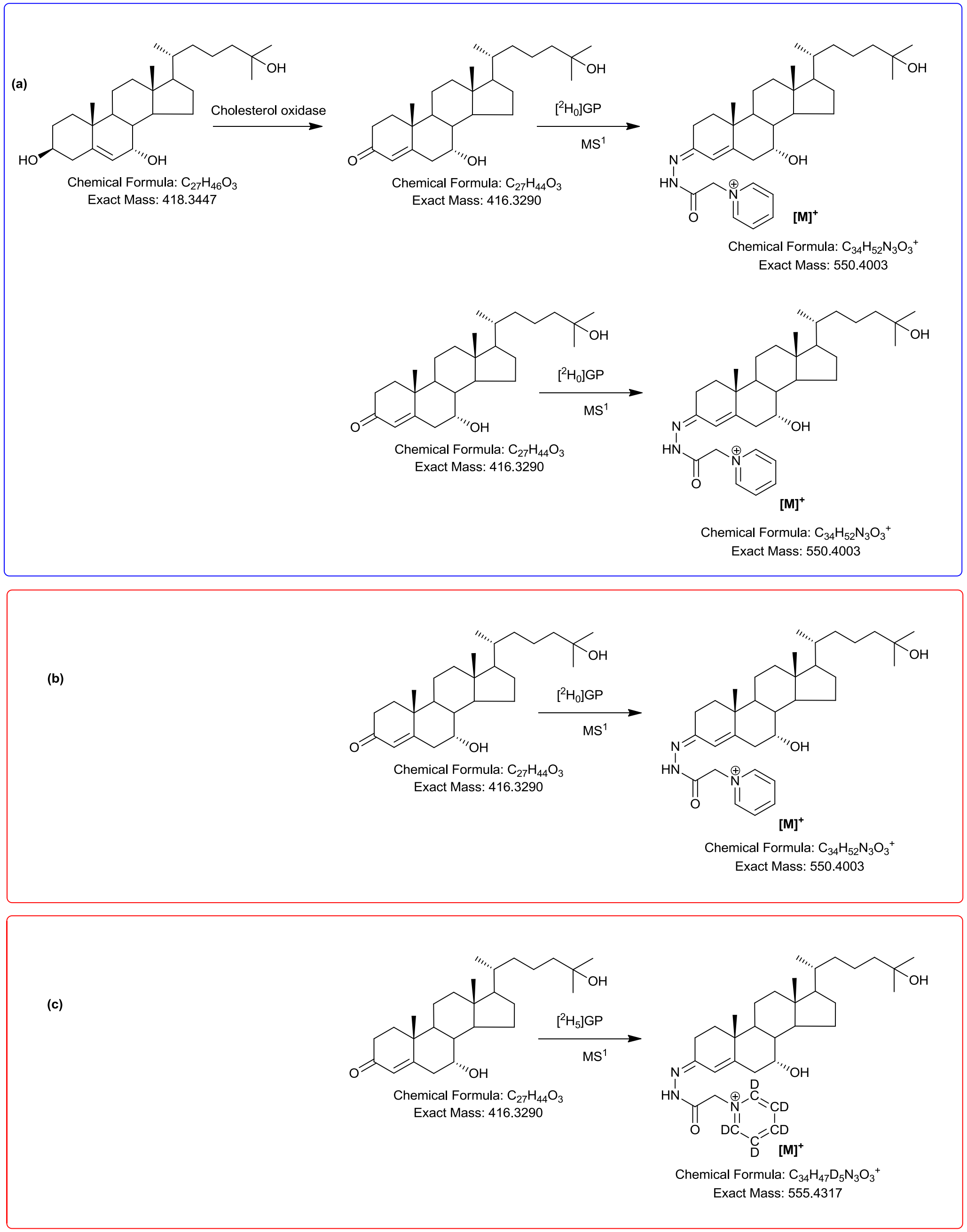

(a)
Cholesterol oxidase
Chemical Formula: C27H46O3
Exact Mass: 418.3447
Chemical Formula: C27H44O3
Exact Mass: 416.3290
[2H0]GP
MS1
[M]+
Chemical Formula: C34H52N3O3+
Exact Mass: 550.4003
Chemical Formula: C27H44O3
Exact Mass: 416.3290
[2H0]GP
MS1
[M]+
Chemical Formula: C34H52N3O3+
Exact Mass: 550.4003
(b)
Chemical Formula: C27H44O3
Exact Mass: 416.3290
[2H0]GP
MS1
[M]+
Chemical Formula: C34H52N3O3+
Exact Mass: 550.4003
(c)
Chemical Formula: C27H44O3
Exact Mass: 416.3290
[2H5]GP
MS1
[M]+
Chemical Formula: C34H47D5N3O3+
Exact Mass: 555.4317

S2

Figure S2

(a)

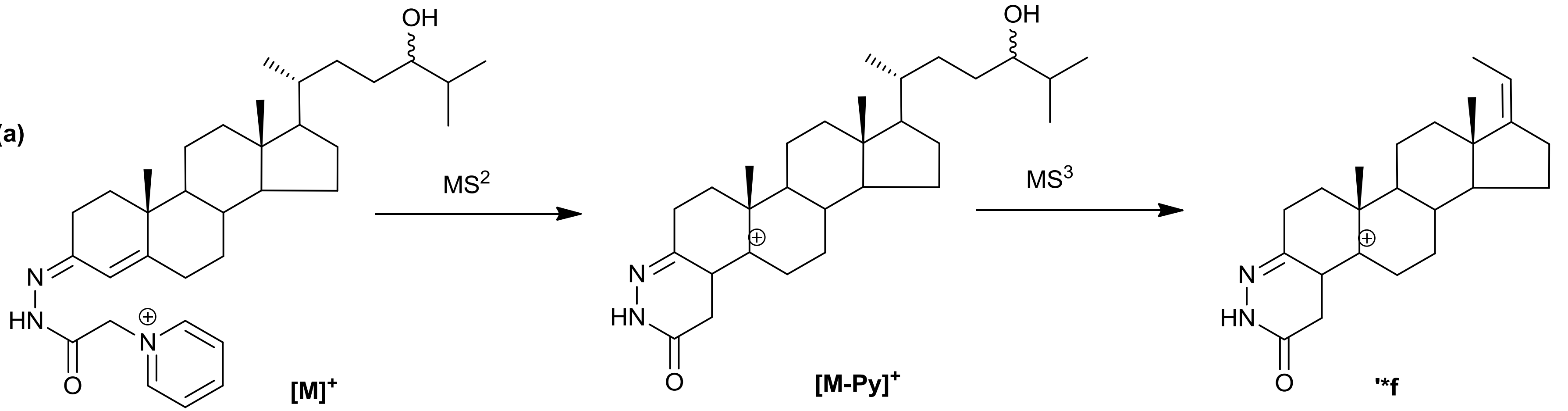

[M]+ — Chemical Formula: $C_{34}H_{52}N_3O_2^+$
Exact Mass: 534.4054

[M-Py]+ — Chemical Formula: $C_{29}H_{47}N_2O_2^+$
Exact Mass: 455.3632

'*f — Chemical Formula: $C_{23}H_{33}N_2O^+$
Exact Mass: 353.2587

S3

Figure S3

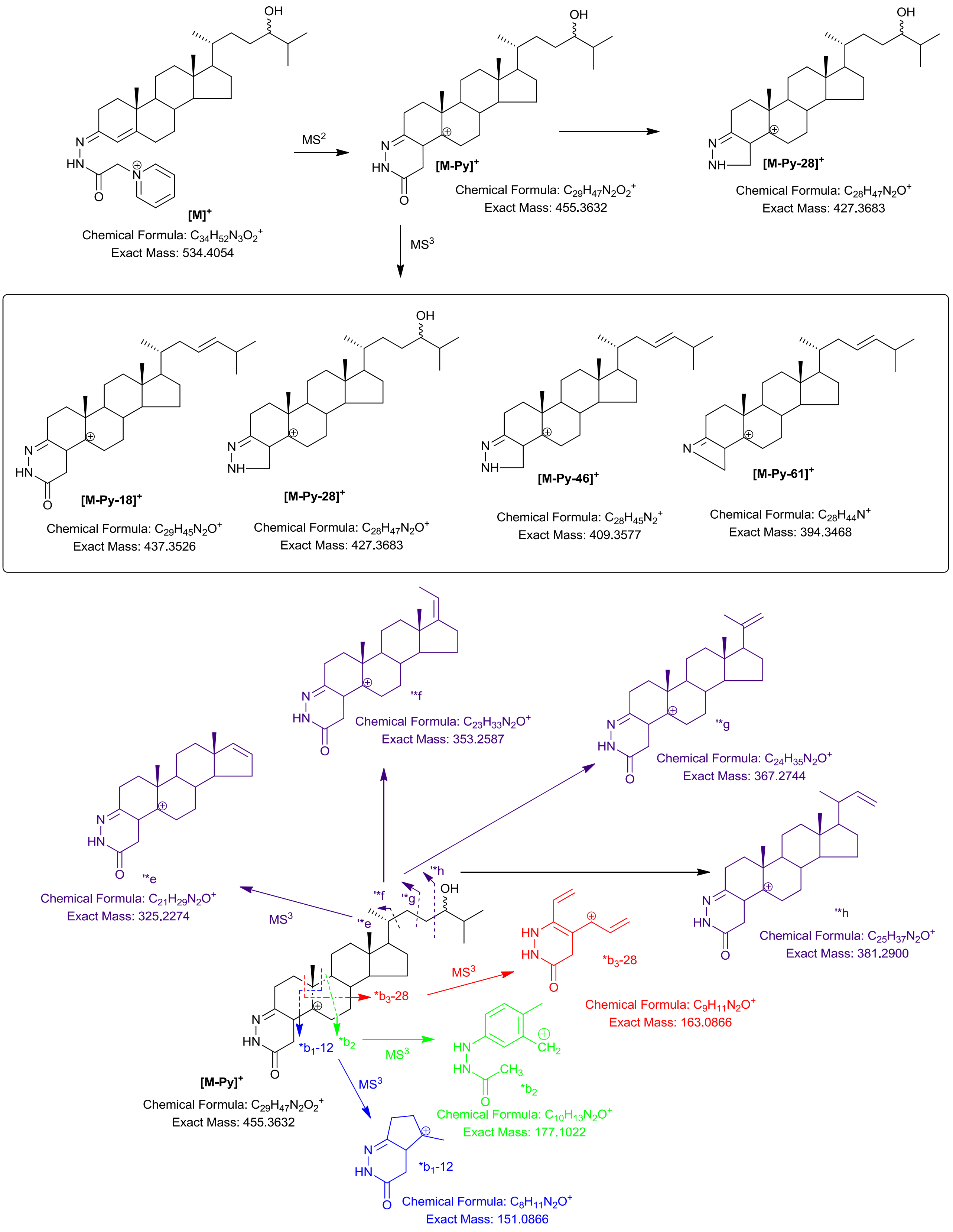

OH
N
HN
O
N
[M]+
Chemical Formula: C34H52N3O2+
Exact Mass: 534.4054
MS2
[M-Py]+
Chemical Formula: C29H47N2O2+
Exact Mass: 455.3632
NH
[M-Py-28]+
Chemical Formula: C28H47N2O+
Exact Mass: 427.3683
MS3
[M-Py-18]+
Chemical Formula: C29H45N2O+
Exact Mass: 437.3526
[M-Py-28]+
Chemical Formula: C28H47N2O+
Exact Mass: 427.3683
[M-Py-46]+
Chemical Formula: C28H45N2+
Exact Mass: 409.3577
[M-Py-61]+
Chemical Formula: C28H44N+
Exact Mass: 394.3468
'*f
Chemical Formula: C23H33N2O+
Exact Mass: 353.2587
'*g
Chemical Formula: C24H35N2O+
Exact Mass: 367.2744
'*e
Chemical Formula: C21H29N2O+
Exact Mass: 325.2274
'*h
Chemical Formula: C25H37N2O+
Exact Mass: 381.2900
*b3-28
Chemical Formula: C9H11N2O+
Exact Mass: 163.0866
*b1-12
*b2
CH2
CH3
Chemical Formula: C10H13N2O+
Exact Mass: 177.1022
Chemical Formula: C8H11N2O+
Exact Mass: 151.0866

S4

Figure S4

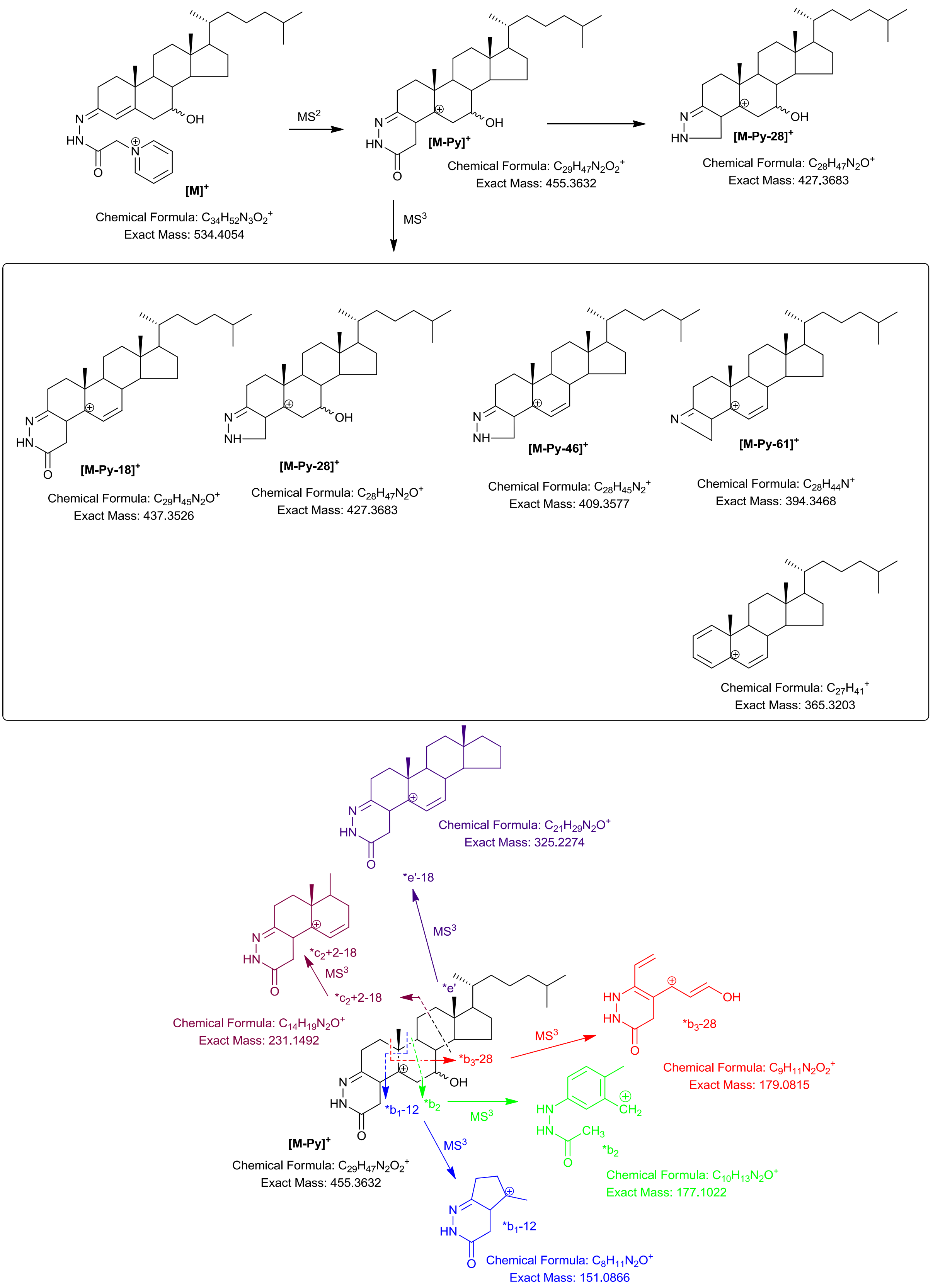

[M]$^+$
Chemical Formula: $C_{34}H_{52}N_3O_2^+$
Exact Mass: 534.4054
MS^2
[M-Py]$^+$
Chemical Formula: $C_{29}H_{47}N_2O_2^+$
Exact Mass: 455.3632
[M-Py-28]$^+$
Chemical Formula: $C_{28}H_{47}N_2O^+$
Exact Mass: 427.3683
MS^3
[M-Py-18]$^+$
Chemical Formula: $C_{29}H_{45}N_2O^+$
Exact Mass: 437.3526
[M-Py-28]$^+$
Chemical Formula: $C_{28}H_{47}N_2O^+$
Exact Mass: 427.3683
[M-Py-46]$^+$
Chemical Formula: $C_{28}H_{45}N_2^+$
Exact Mass: 409.3577
[M-Py-61]$^+$
Chemical Formula: $C_{28}H_{44}N^+$
Exact Mass: 394.3468
Chemical Formula: $C_{27}H_{41}^+$
Exact Mass: 365.3203
Chemical Formula: $C_{21}H_{29}N_2O^+$
Exact Mass: 325.2274
*e'-18
MS^3
*c$_2$+2-18
MS^3
*c$_2$+2-18
Chemical Formula: $C_{14}H_{19}N_2O^+$
Exact Mass: 231.1492
*e'
*b$_3$-28
MS^3
*b$_3$-28
Chemical Formula: $C_9H_{11}N_2O_2^+$
Exact Mass: 179.0815
*b$_1$-12
*b$_2$
MS^3
*b$_2$
Chemical Formula: $C_{10}H_{13}N_2O^+$
Exact Mass: 177.1022
[M-Py]$^+$
Chemical Formula: $C_{29}H_{47}N_2O_2^+$
Exact Mass: 455.3632
MS^3
*b$_1$-12
Chemical Formula: $C_8H_{11}N_2O^+$
Exact Mass: 151.0866

S5a

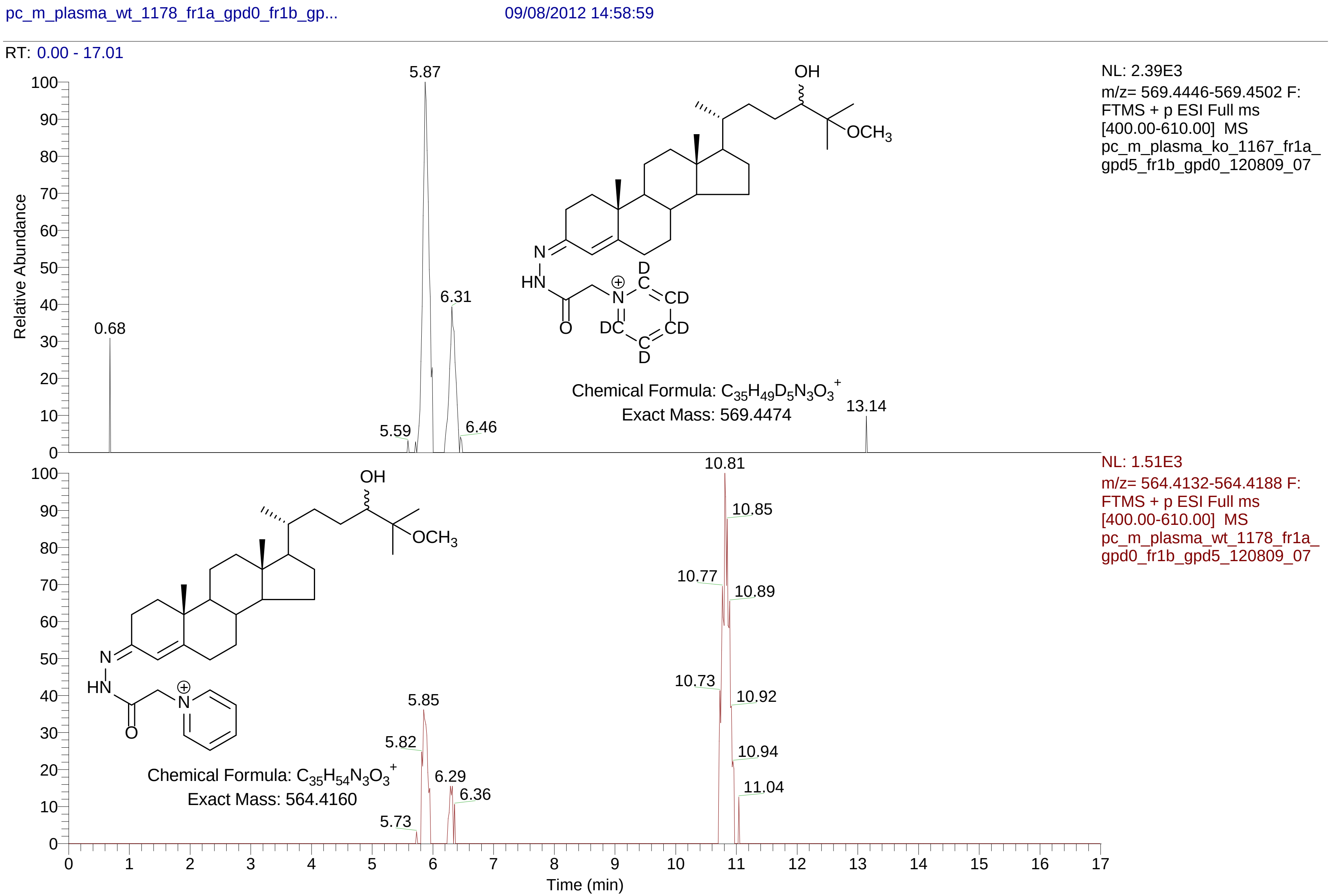
pc_m_plasma_wt_1178_fr1a_gpd0_fr1b_gp...
09/08/2012 14:58:59
RT: 0.00 - 17.01
NL: 2.39E3
m/z= 569.4446-569.4502 F: FTMS + p ESI Full ms [400.00-610.00] MS pc_m_plasma_ko_1167_fr1a_gpd5_fr1b_gpd0_120809_07
5.87
6.31
0.68
5.59
6.46
13.14
OH
OCH3
N
HN
O
D
C
CD
DC
CD
C
D
N
Chemical Formula: C35H49D5N3O3+
Exact Mass: 569.4474
NL: 1.51E3
m/z= 564.4132-564.4188 F: FTMS + p ESI Full ms [400.00-610.00] MS pc_m_plasma_wt_1178_fr1a_gpd0_fr1b_gpd5_120809_07
10.81
10.85
10.77
10.89
10.73
10.92
10.94
11.04
5.85
5.82
6.29
6.36
5.73
OH
OCH3
N
HN
O
N
Chemical Formula: C35H54N3O3+
Exact Mass: 564.4160
Relative Abundance
Time (min)

S5b

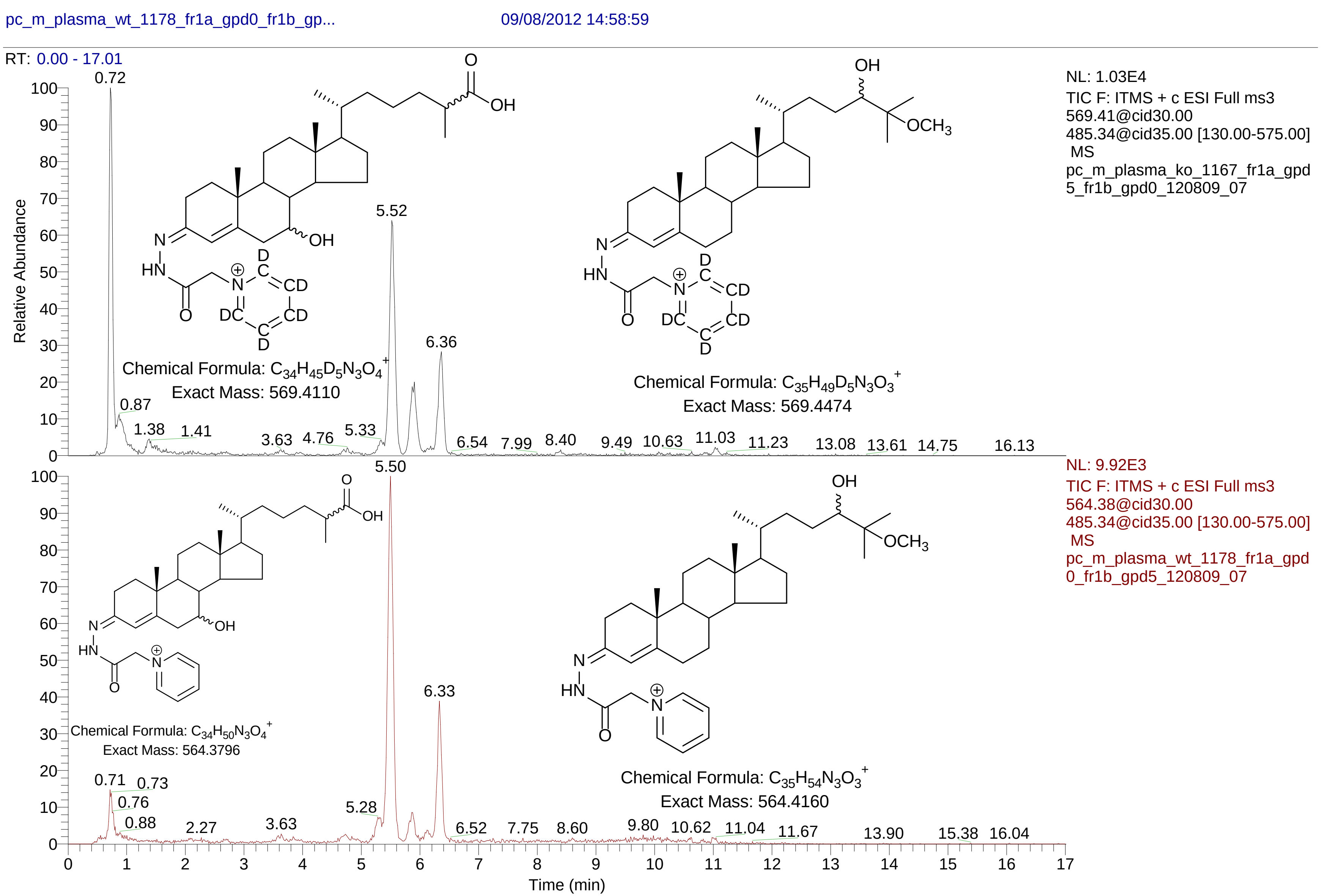

pc_m_plasma_wt_1178_fr1a_gpd0_fr1b_gp...
09/08/2012 14:58:59
RT: 0.00 - 17.01
NL: 1.03E4
TIC F: ITMS + c ESI Full ms3 569.41@cid30.00 485.34@cid35.00 [130.00-575.00] MS
pc_m_plasma_ko_1167_fr1a_gpd5_fr1b_gpd0_120809_07
Chemical Formula: C34H45D5N3O4+
Exact Mass: 569.4110
Chemical Formula: C35H49D5N3O3+
Exact Mass: 569.4474
NL: 9.92E3
TIC F: ITMS + c ESI Full ms3 564.38@cid30.00 485.34@cid35.00 [130.00-575.00] MS
pc_m_plasma_wt_1178_fr1a_gpd0_fr1b_gpd5_120809_07
Chemical Formula: C34H50N3O4+
Exact Mass: 564.3796
Chemical Formula: C35H54N3O3+
Exact Mass: 564.4160
Relative Abundance
Time (min)

S5c

pc_m_plasma_ko_1167_fr1a_gpd5_fr1b_gp... 10/08/2012 03:59:44

pc_m_plasma_ko_1167_fr1a_gpd5_fr1b_gpd0_120809_07 #1158 RT: 5.87 AV: 1 NL: 1.30E3
F: ITMS + c ESI Full ms3 569.41@cid30.00 485.34@cid35.00 [130.00-575.00]

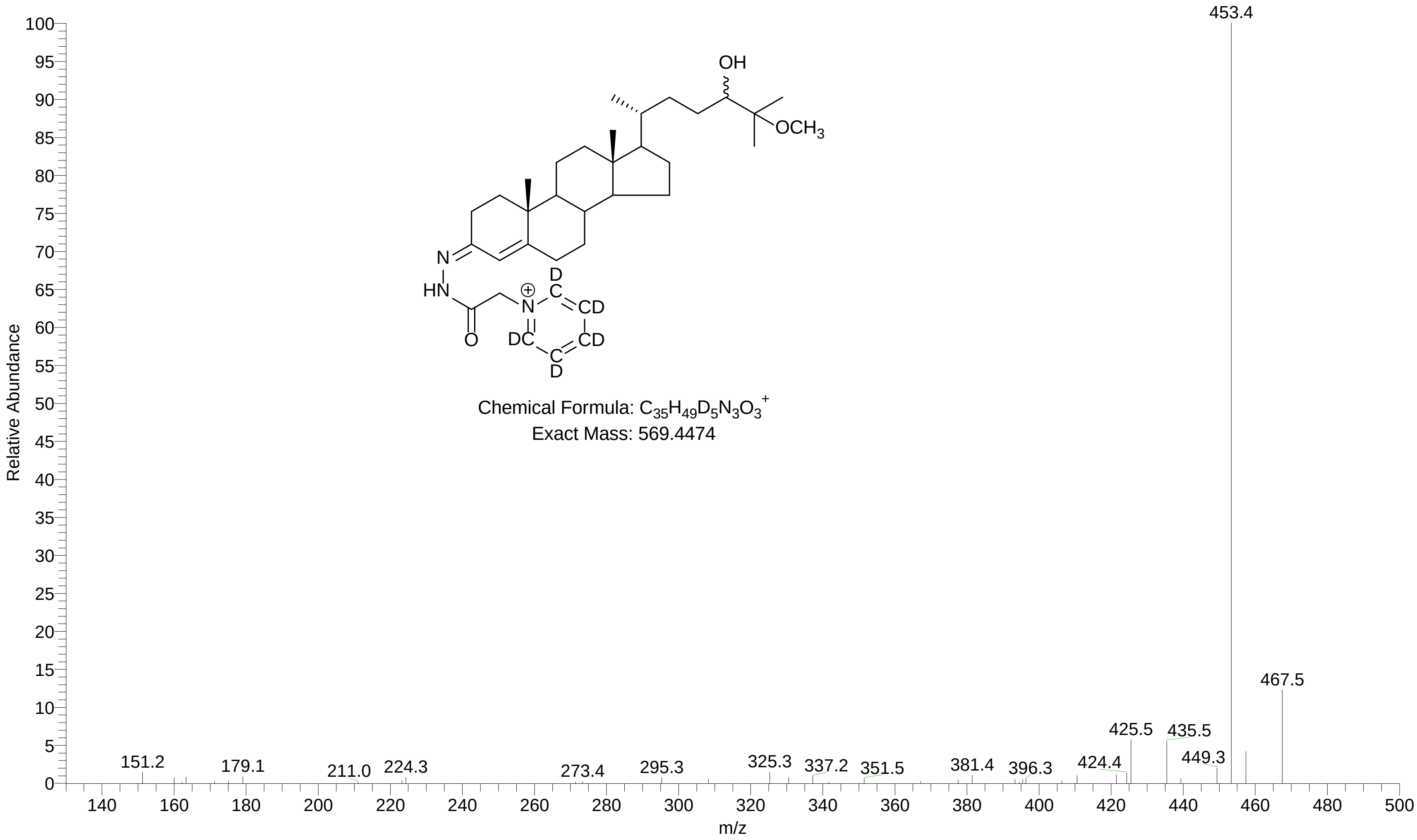

S6a

pc_m_plasma_ko_1167_fr1a_gpd5_fr1b_gp... 10/08/2012 02:25:14

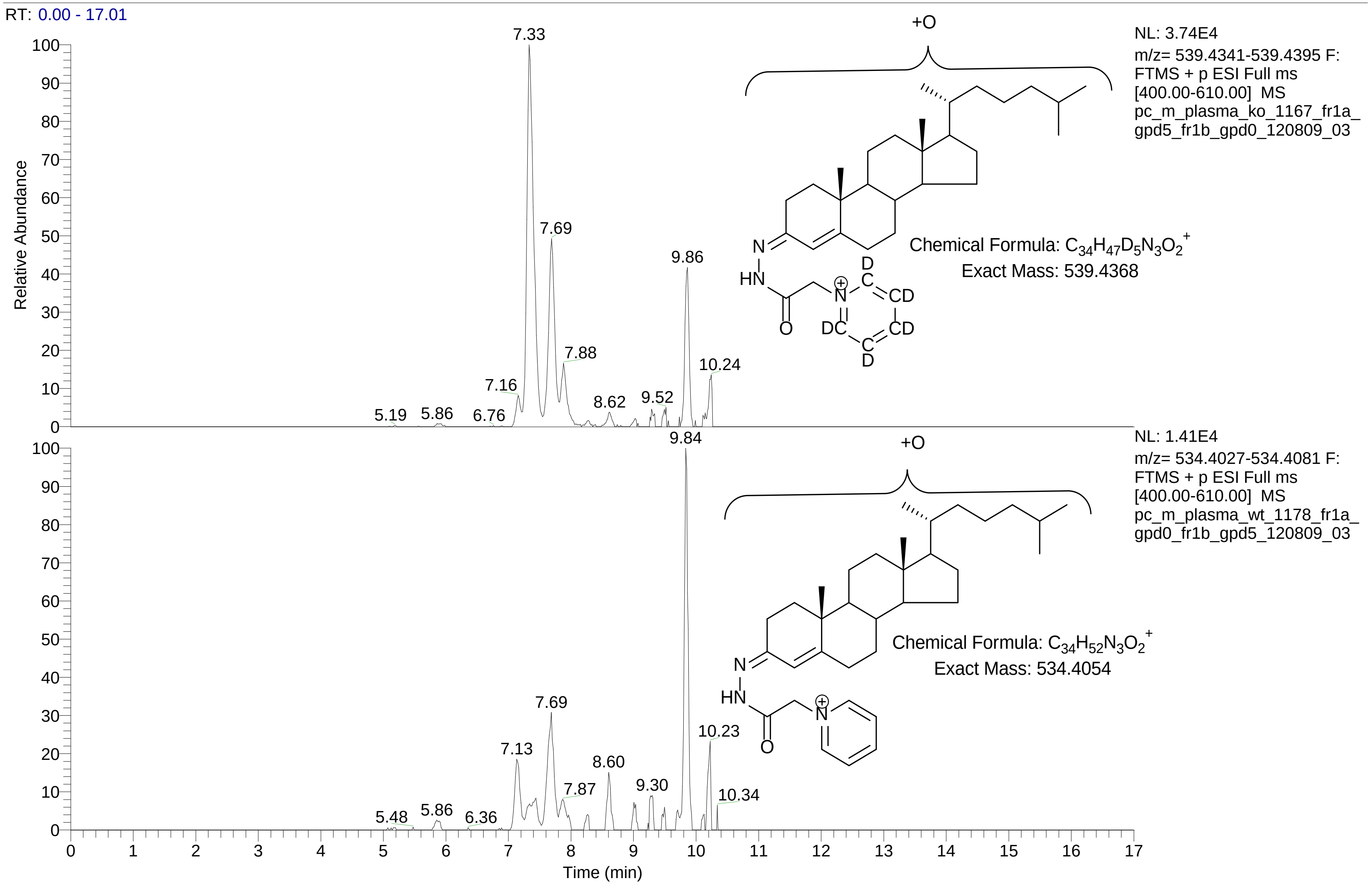

S6b

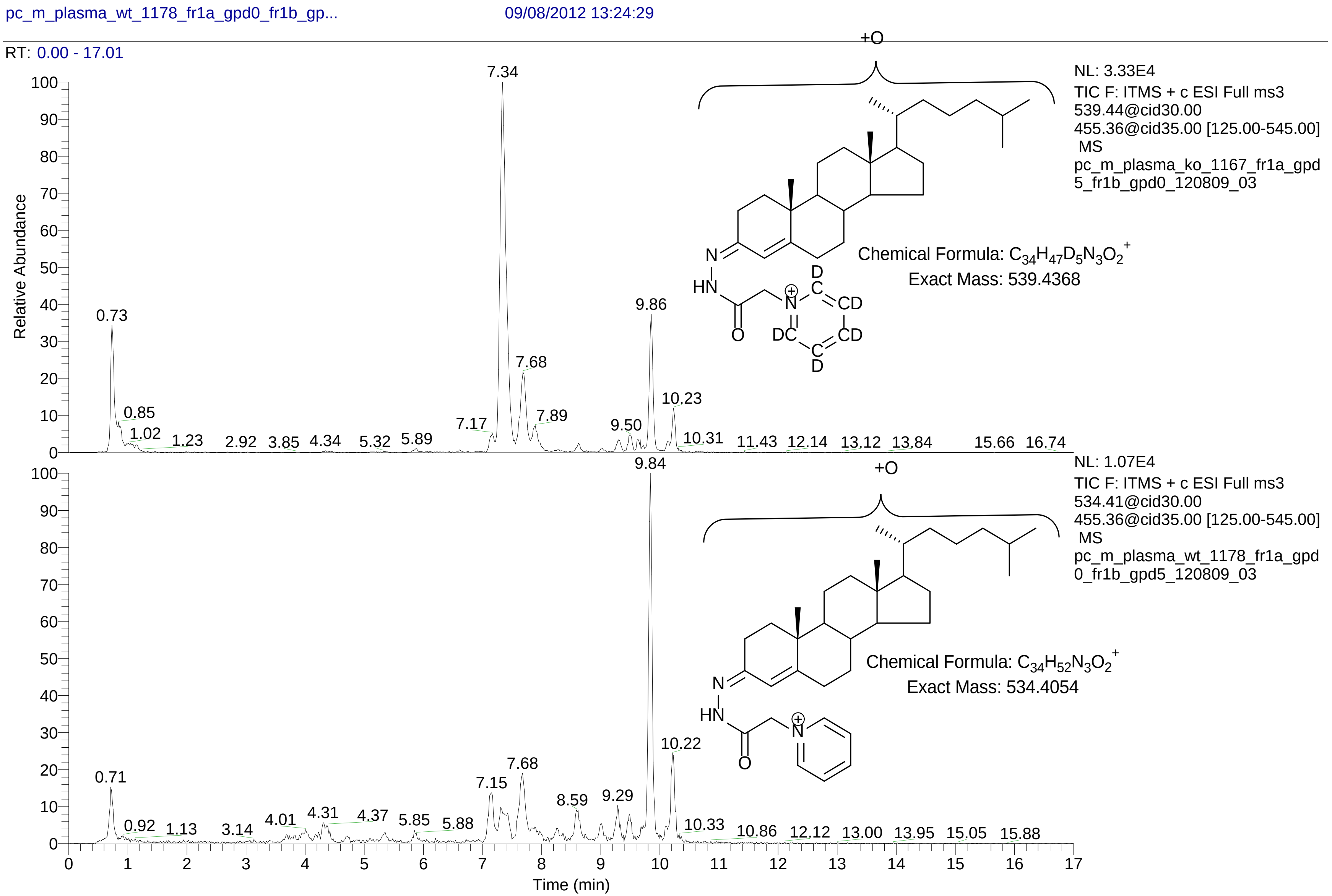

pc_m_plasma_wt_1178_fr1a_gpd0_fr1b_gp...
09/08/2012 13:24:29
RT: 0.00 - 17.01
NL: 3.33E4
TIC F: ITMS + c ESI Full ms3 539.44@cid30.00 455.36@cid35.00 [125.00-545.00] MS pc_m_plasma_ko_1167_fr1a_gpd5_fr1b_gpd0_120809_03
+O
Chemical Formula: C34H47D5N3O2+
Exact Mass: 539.4368
NL: 1.07E4
TIC F: ITMS + c ESI Full ms3 534.41@cid30.00 455.36@cid35.00 [125.00-545.00] MS pc_m_plasma_wt_1178_fr1a_gpd0_fr1b_gpd5_120809_03
Chemical Formula: C34H52N3O2+
Exact Mass: 534.4054
Relative Abundance
Time (min)

S6c

PC_m_plasma_KO_1167_Fr1a_GPd5_Fr1b_GP... 10/08/2012 02:25:14

PC_m_plasma_KO_1167_Fr1a_GPd5_Fr1b_GPd0_120809_03 #1152-1159 RT: 5.84-5.87 AV: 3 NL: 3.70E1
F: ITMS + c ESI Full ms3 539.44@cid30.00 455.36@cid35.00 [125.00-545.00]

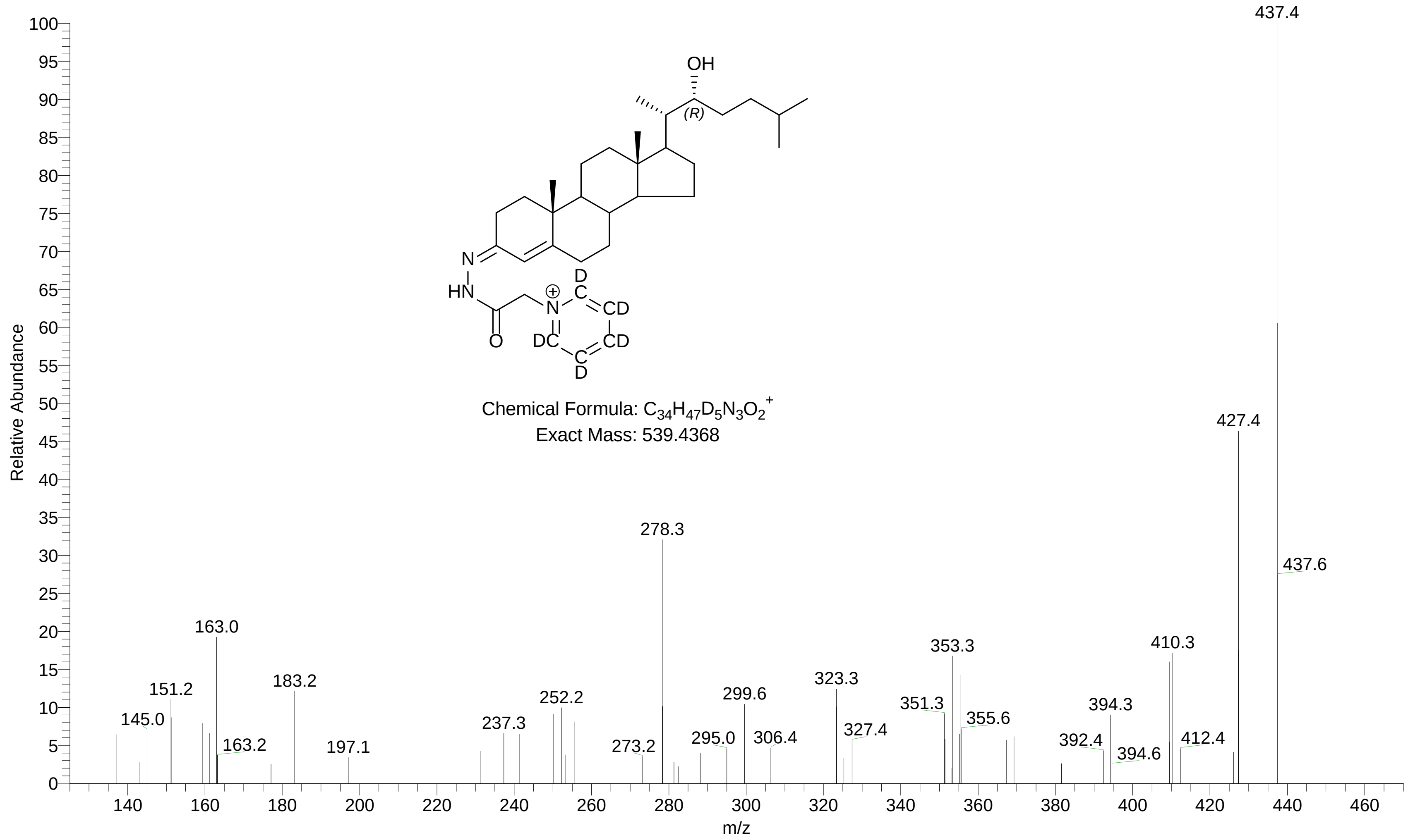

S6d

PC_m_plasma_KO_1167_Fr1a_GPd5_Fr1b_GP... 08/10/12 02:25:14

PC_m_plasma_KO_1167_Fr1a_GPd5_Fr1b_GPd0_120809_03 #1386 RT: 7.15 AV: 1 NL: 5.19E2
F: ITMS + c ESI Full ms3 539.44@cid30.00 455.36@cid35.00 [125.00-545.00]

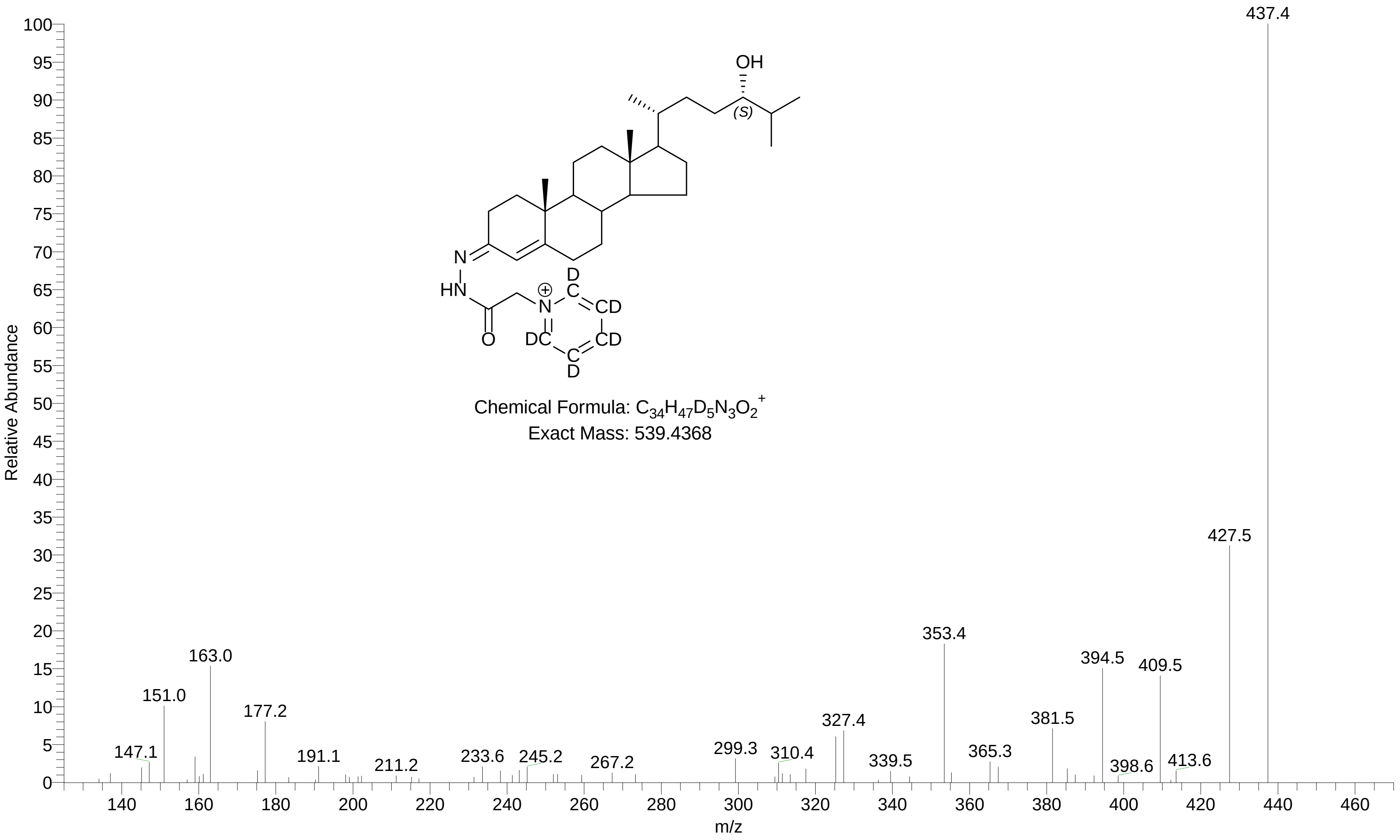

S6e

PC_m_plasma_KO_1167_Fr1a_GPd5_Fr1b_GP... 08/10/12 02:25:14

PC_m_plasma_KO_1167_Fr1a_GPd5_Fr1b_GPd0_120809_03 #1419 RT: 7.34 AV: 1 NL: 2.77E4
F: ITMS + c ESI Full ms3 539.44@cid30.00 455.36@cid35.00 [125.00-545.00]

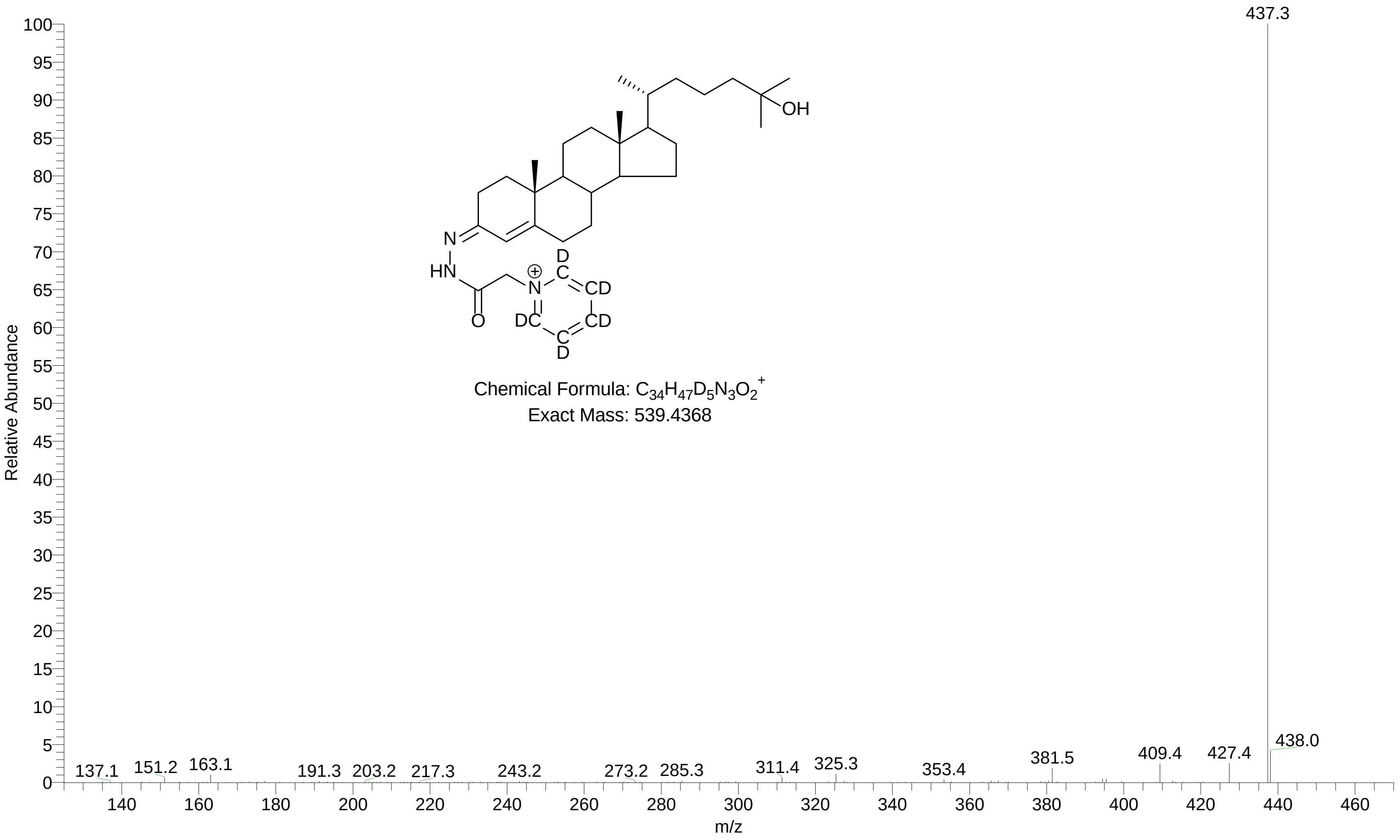

S6f

PC_m_plasma_KO_1167_Fr1a_GPd5_Fr1b_GP... 08/10/12 02:25:14

PC_m_plasma_KO_1167_Fr1a_GPd5_Fr1b_GPd0_120809_03 #1482 RT: 7.68 AV: 1 NL: 1.91E3
F: ITMS + c ESI Full ms3 539.44@cid30.00 455.36@cid35.00 [125.00-545.00]

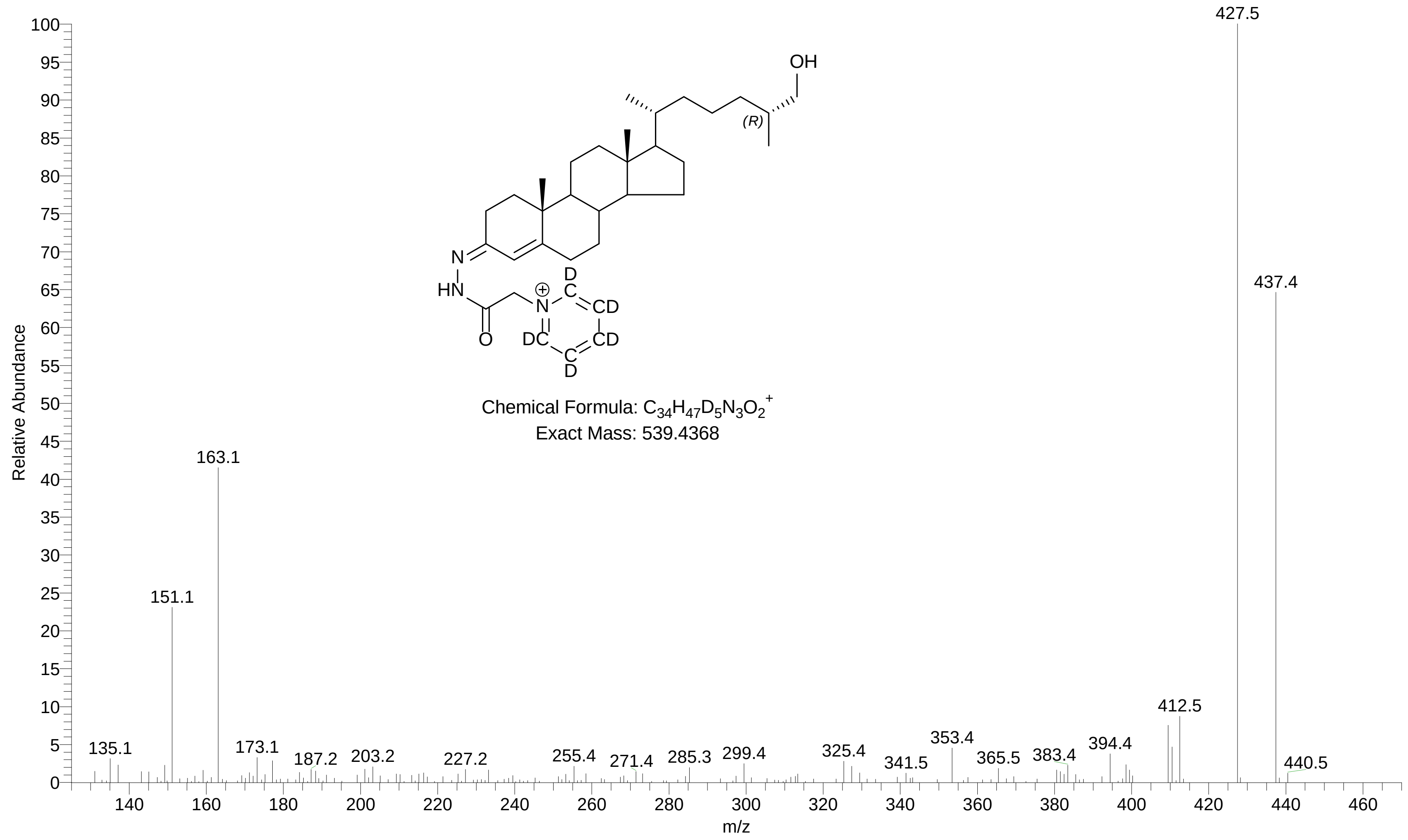

S6g

PC_m_plasma_KO_1167_Fr1a_GPd5_Fr1b_GP... 08/10/12 02:25:14

PC_m_plasma_KO_1167_Fr1a_GPd5_Fr1b_GPd0_120809_03 #1812 RT: 9.30 AV: 1 NL: 3.84E2
F: ITMS + c ESI Full ms3 539.44@cid30.00 455.36@cid35.00 [125.00-545.00]

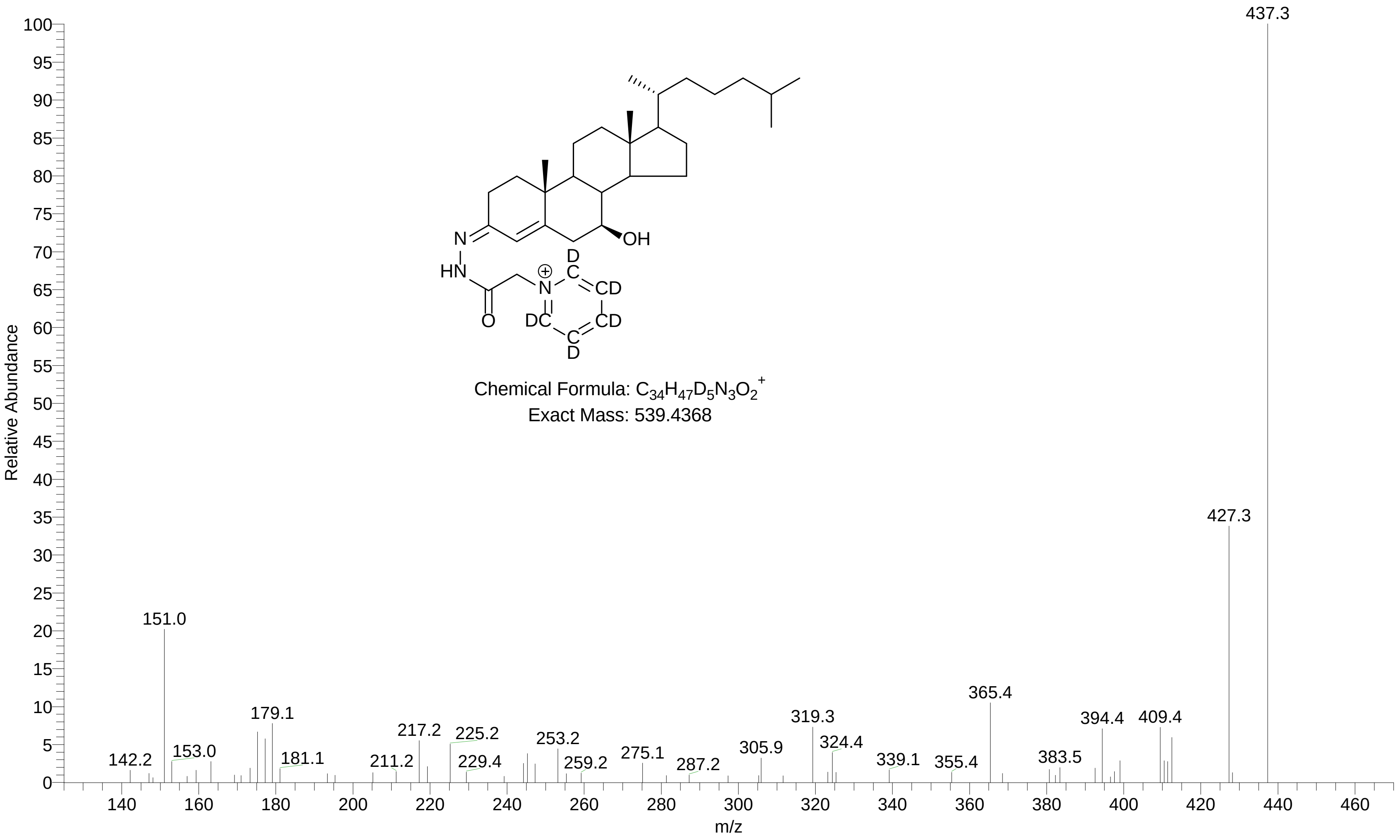

S6h

PC_m_plasma_KO_1167_Fr1a_GPd5_Fr1b_GP... 08/10/12 02:25:14

PC_m_plasma_KO_1167_Fr1a_GPd5_Fr1b_GPd0_120809_03 #1953 RT: 9.86 AV: 1 NL: 4.76E3
F: ITMS + c ESI Full ms3 539.44@cid30.00 455.36@cid35.00 [125.00-545.00]

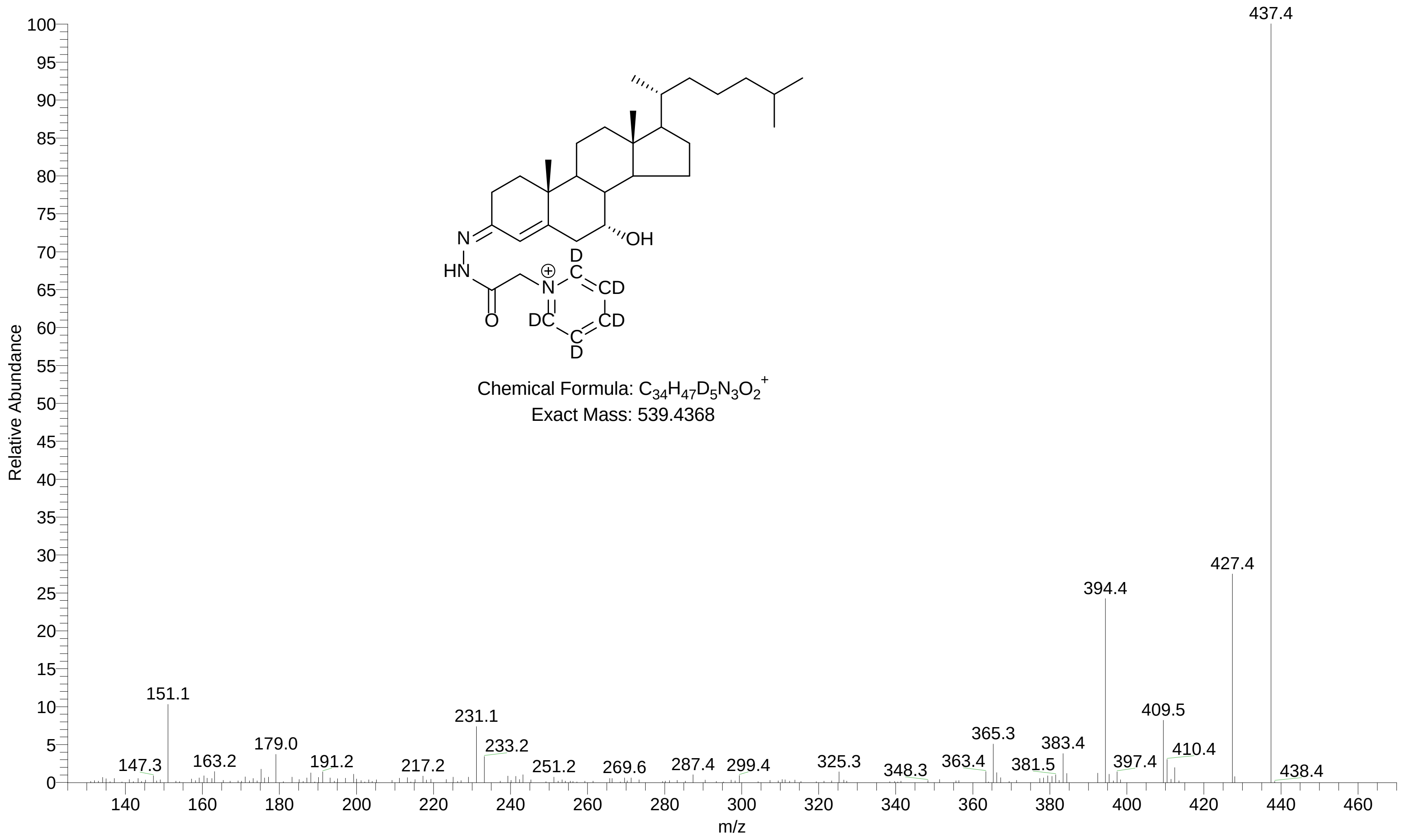

S7a

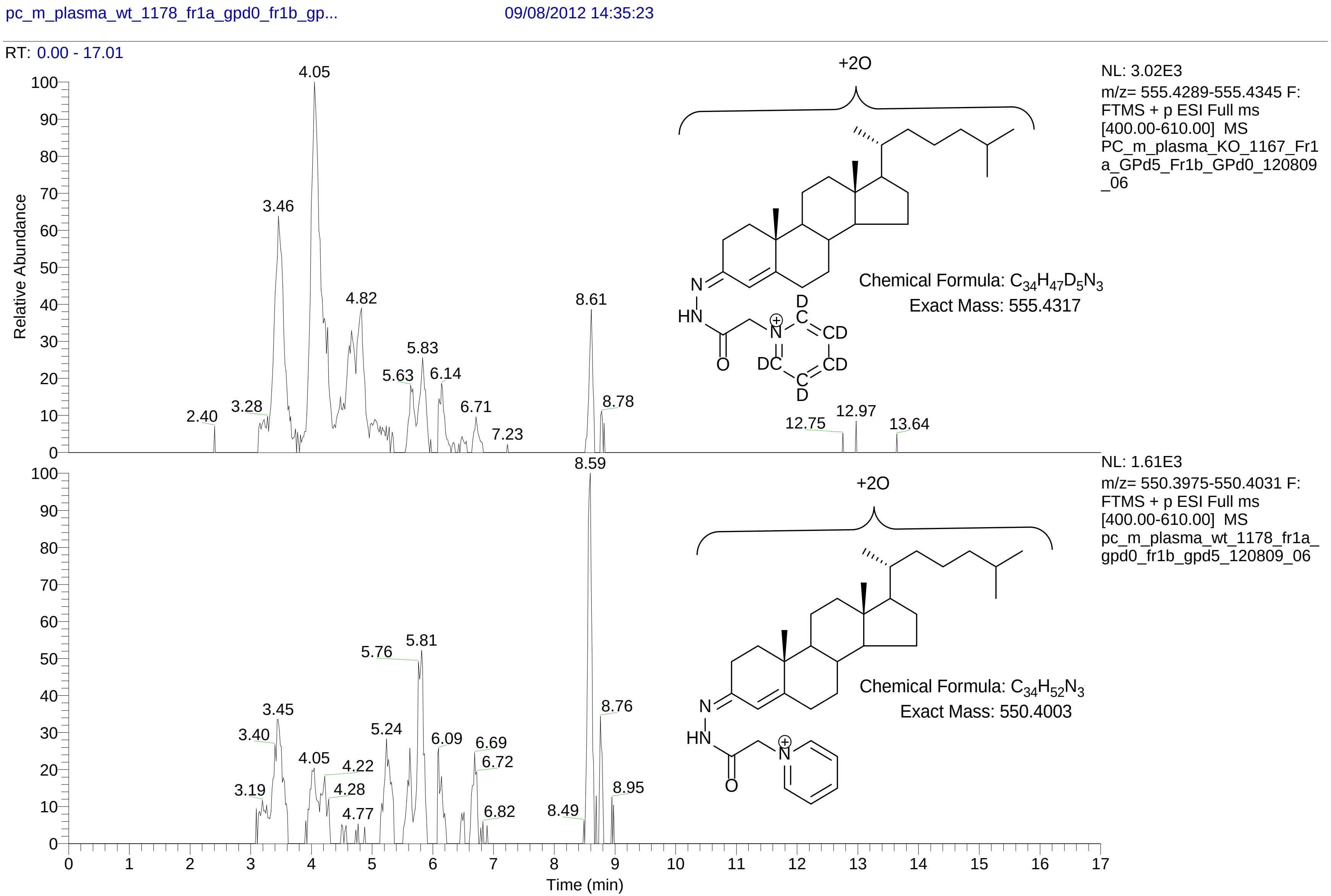

pc_m_plasma_wt_1178_fr1a_gpd0_fr1b_gp...
09/08/2012 14:35:23
RT: 0.00 - 17.01
NL: 3.02E3
m/z= 555.4289-555.4345 F: FTMS + p ESI Full ms [400.00-610.00] MS PC_m_plasma_KO_1167_Fr1a_GPd5_Fr1b_GPd0_120809_06
+2O
Chemical Formula: $C_{34}H_{47}D_5N_3$
Exact Mass: 555.4317
4.05
3.46
4.82
8.61
5.83
5.63
6.14
8.78
6.71
3.28
2.40
7.23
12.97
12.75
13.64
NL: 1.61E3
m/z= 550.3975-550.4031 F: FTMS + p ESI Full ms [400.00-610.00] MS pc_m_plasma_wt_1178_fr1a_gpd0_fr1b_gpd5_120809_06
+2O
Chemical Formula: $C_{34}H_{52}N_3$
Exact Mass: 550.4003
8.59
5.81
5.76
8.76
3.45
3.40
5.24
6.09
6.69
4.05
4.22
6.72
3.19
4.28
8.95
4.77
6.82
8.49
Relative Abundance
Time (min)

S7b

PC_m_plasma_KO_1167_Fr1a_GPd5_Fr1b_GP... 10/08/2012 03:36:10

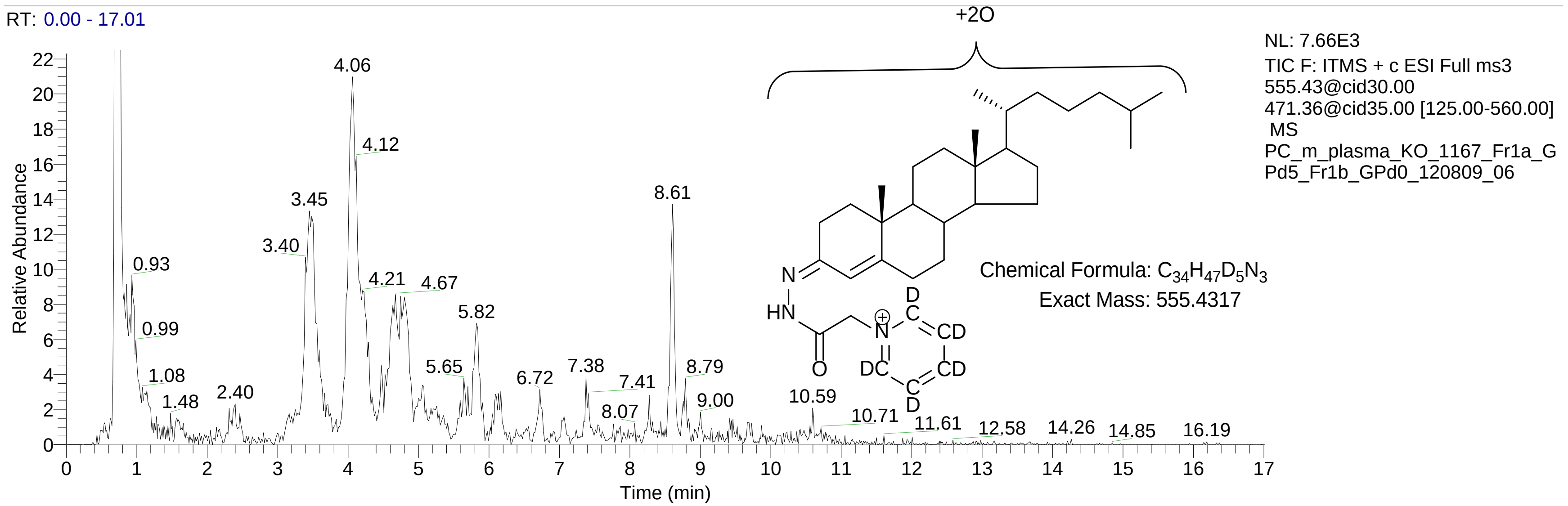

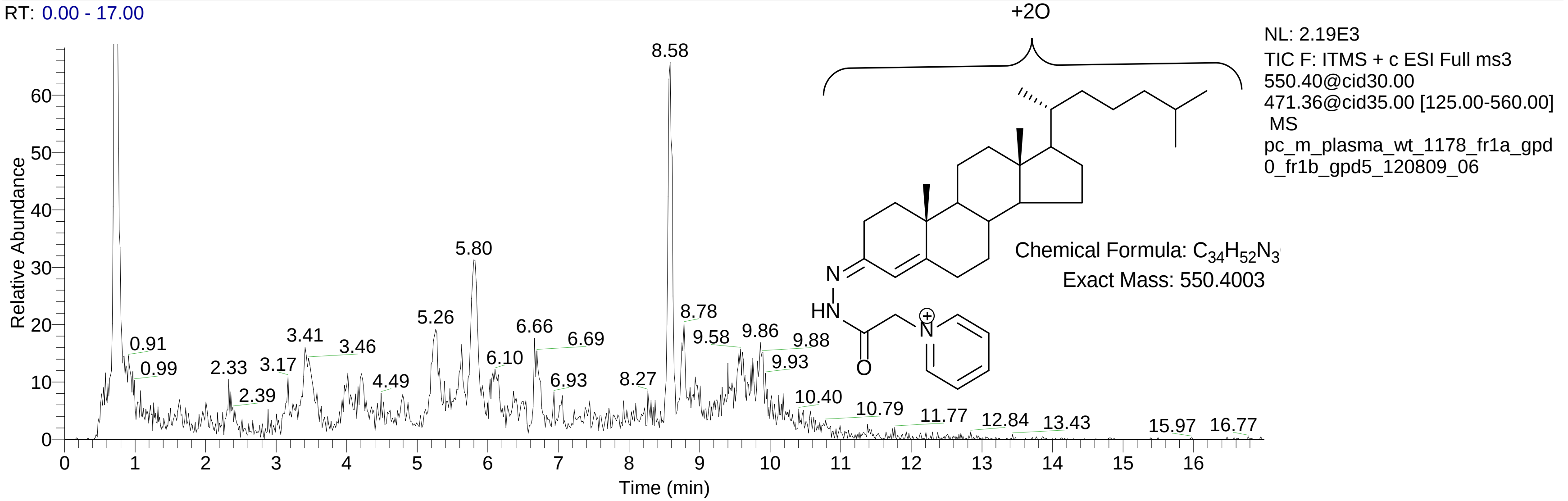

S7c

PC_m_plasma_KO_1167_Fr1a_GPd5_Fr1b_GP... 10/08/2012 03:36:10

PC_m_plasma_KO_1167_Fr1a_GPd5_Fr1b_GPd0_120809_06 #735 RT: 3.47 AV: 1 NL: 5.25E2
F: ITMS + c ESI Full ms3 555.43@cid30.00 471.36@cid35.00 [125.00-560.00]

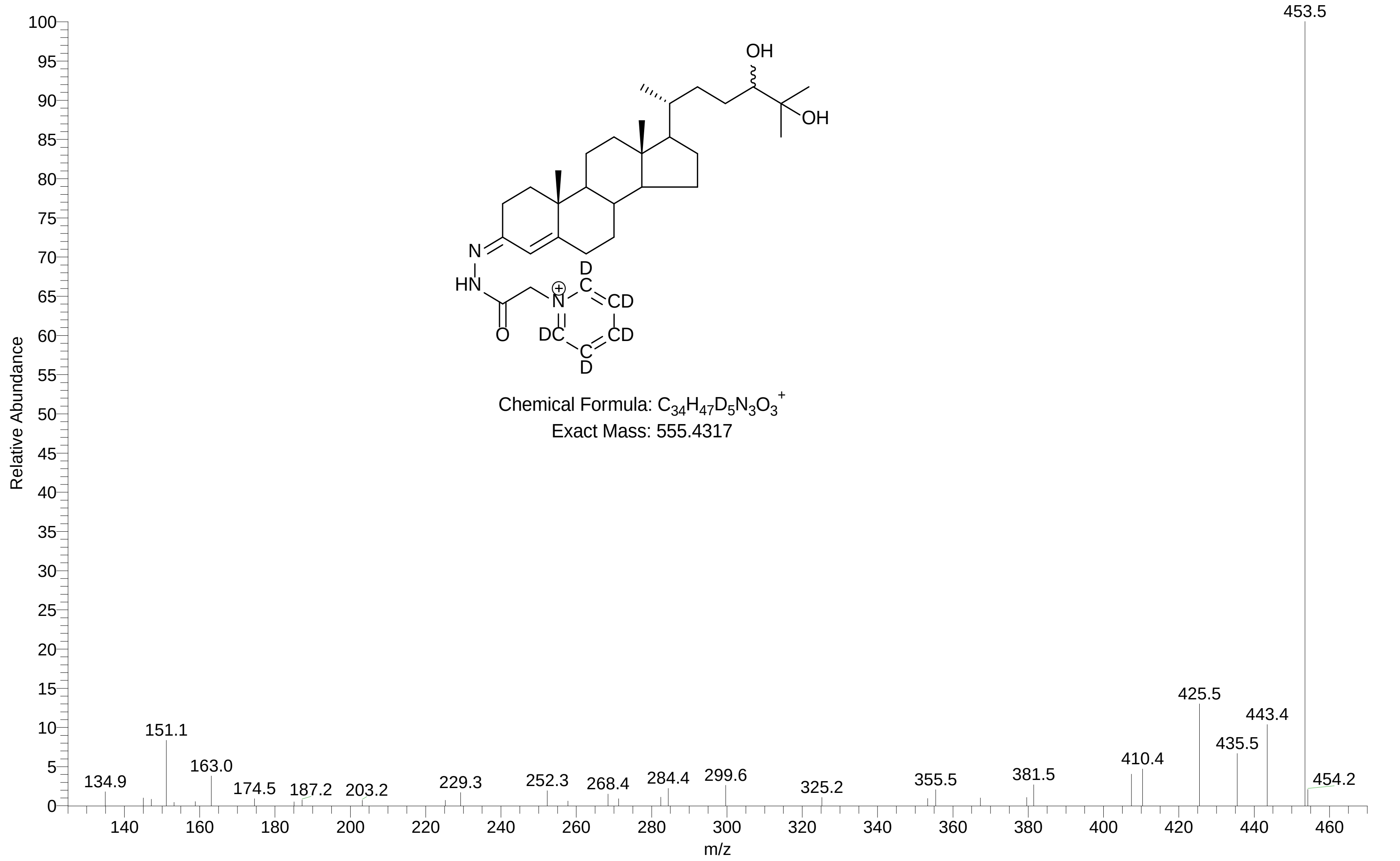

S7d

pc_m_plasma_wt_1178_fr1a_gpd0_fr1b_gp... 09/08/2012 14:35:23

pc_m_plasma_wt_1178_fr1a_gpd0_fr1b_gpd5_120809_06 #1061 RT: 5.26 AV: 1 NL: 2.10E2
F: ITMS + c ESI Full ms3 550.40@cid30.00 471.36@cid35.00 [125.00-560.00]

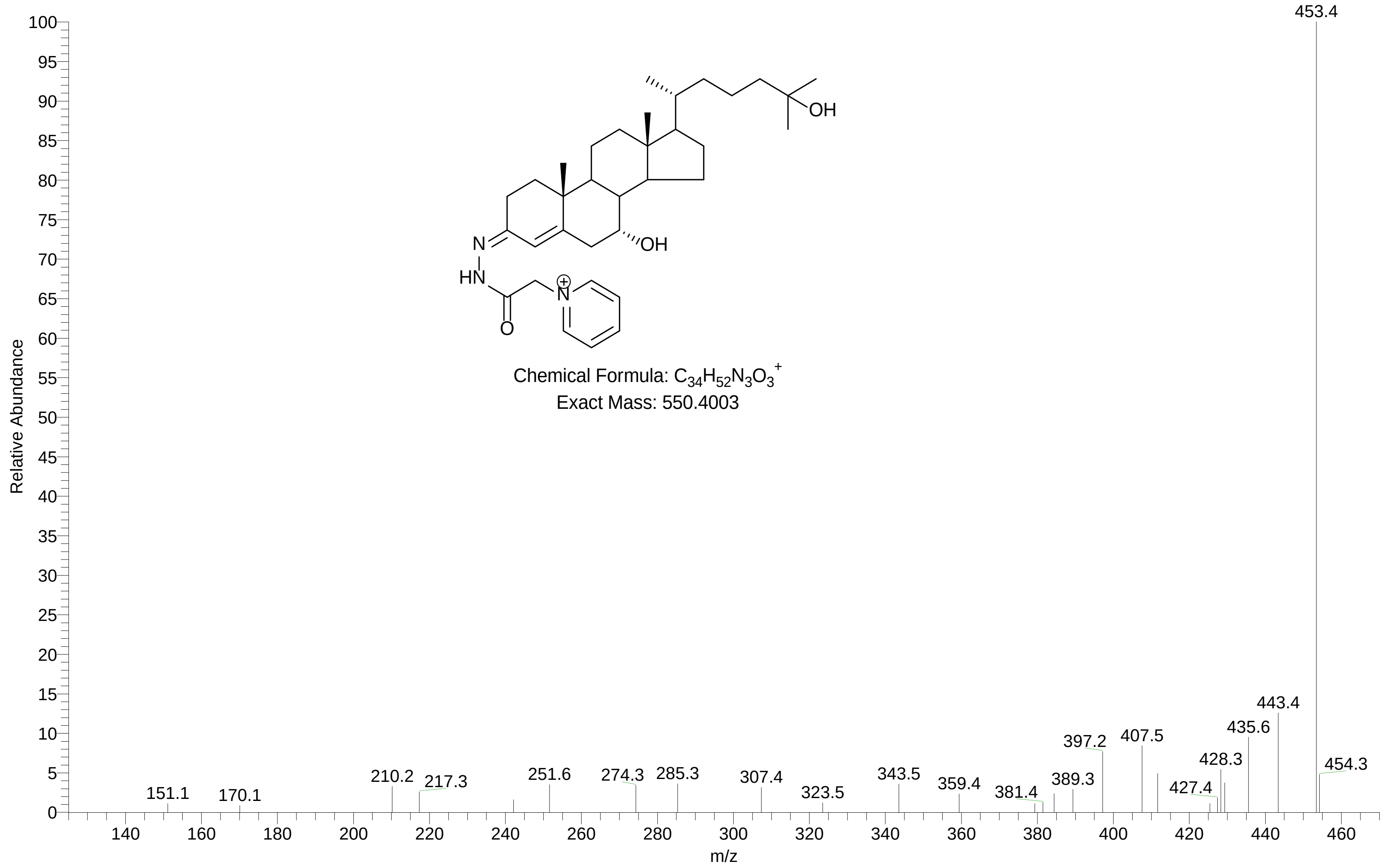

S7e

pc_m_plasma_wt_1178_fr1a_gpd0_fr1b_gp... 09/08/2012 14:35:23

pc_m_plasma_wt_1178_fr1a_gpd0_fr1b_gpd5_120809_06 #1152-1165 RT: 5.77-5.82 AV: 4 NL: 7.36E1
F: ITMS + c ESI Full ms3 550.40@cid30.00 471.36@cid35.00 [125.00-560.00]

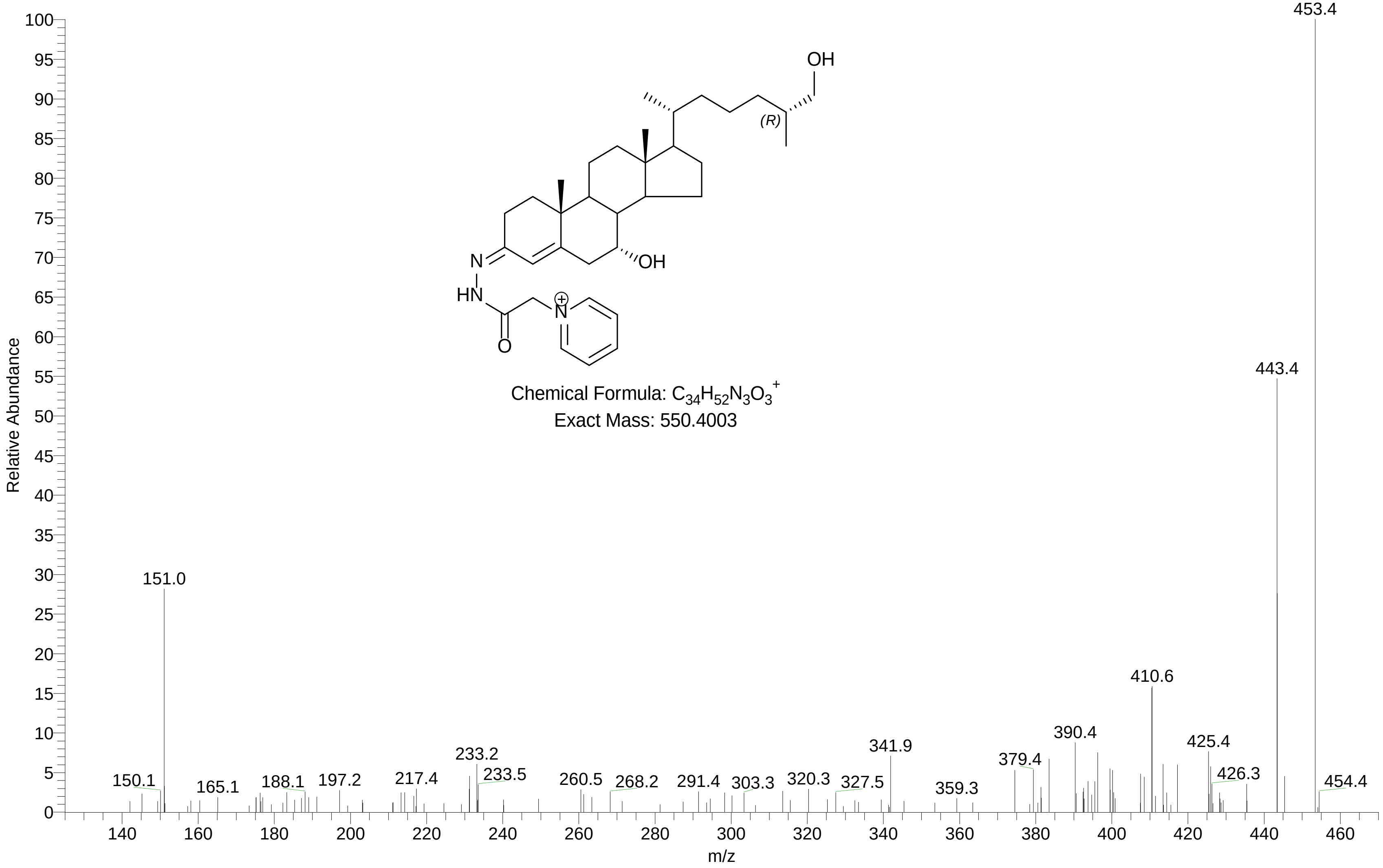

S7f

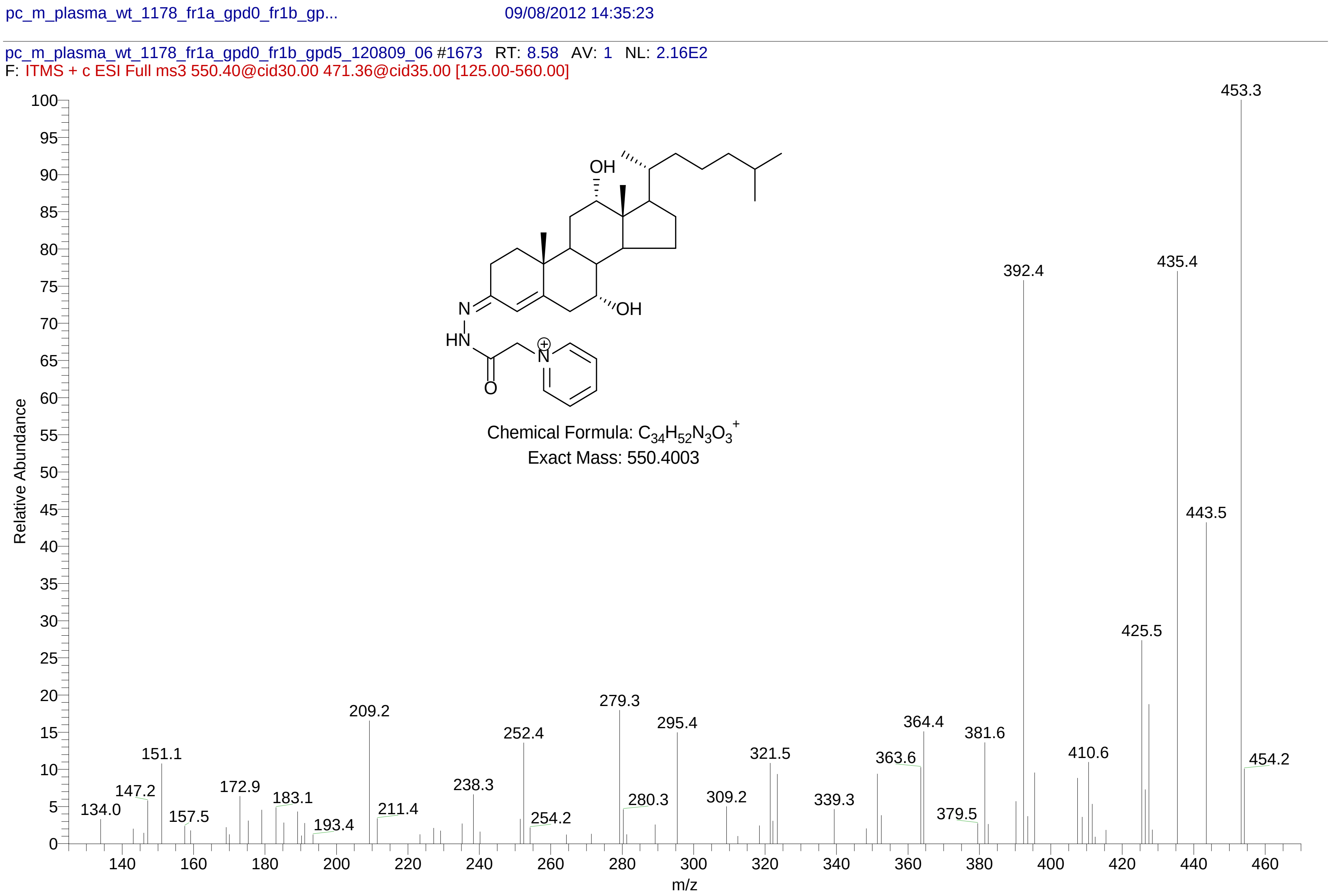

pc_m_plasma_wt_1178_fr1a_gpd0_fr1b_gp...
09/08/2012 14:35:23
pc_m_plasma_wt_1178_fr1a_gpd0_fr1b_gpd5_120809_06 #1673 RT: 8.58 AV: 1 NL: 2.16E2
F: ITMS + c ESI Full ms3 550.40@cid30.00 471.36@cid35.00 [125.00-560.00]
Relative Abundance
m/z
453.3
392.4
435.4
443.5
425.5
209.2
279.3
295.4
252.4
364.4
381.6
151.1
321.5
363.6
410.6
454.2
147.2
172.9
238.3
183.1
134.0
157.5
193.4
211.4
254.2
280.3
309.2
339.3
379.5
OH
OH
N
HN
O
N
Chemical Formula: C34H52N3O3+
Exact Mass: 550.4003

S8a

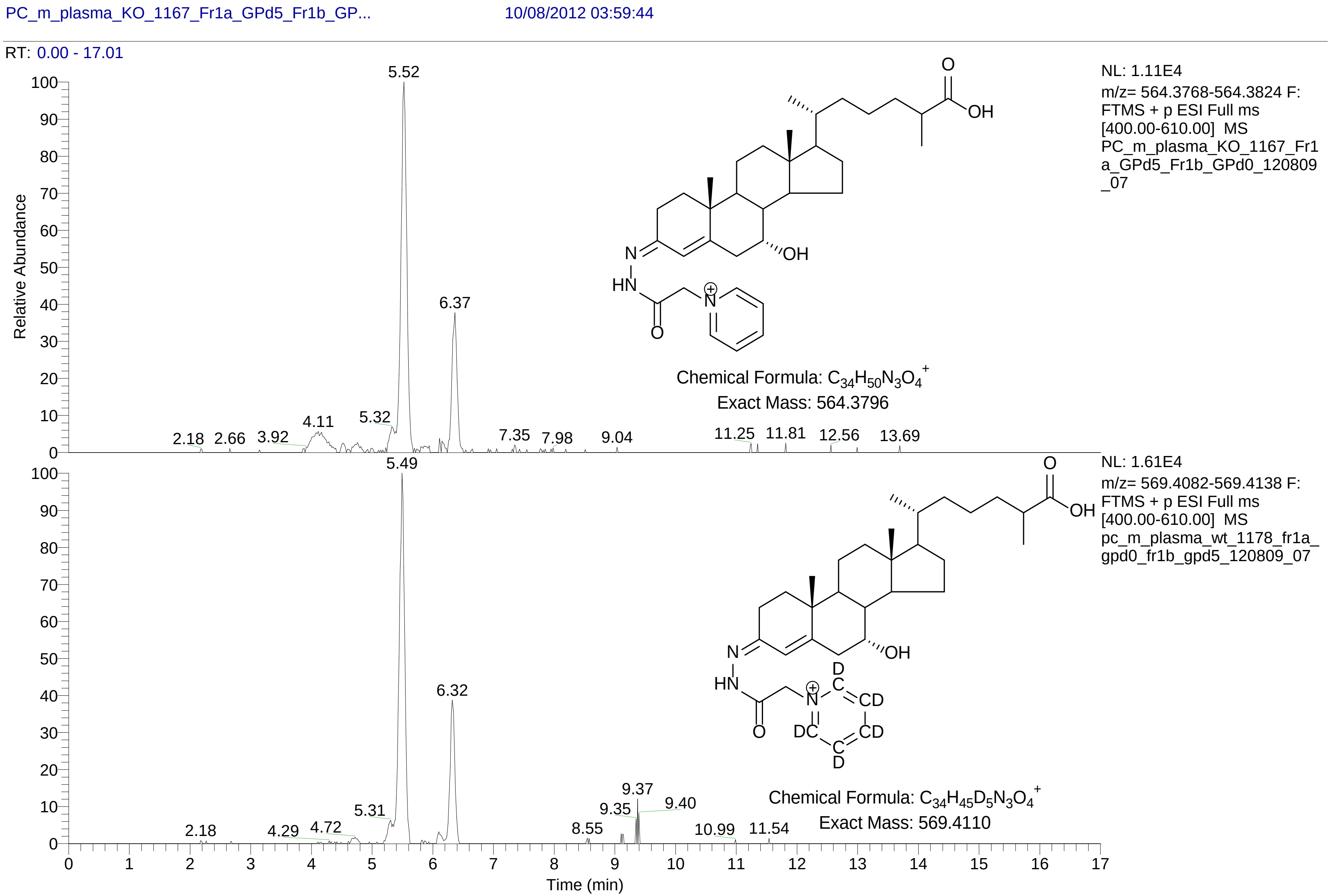

PC_m_plasma_KO_1167_Fr1a_GPd5_Fr1b_GP...
10/08/2012 03:59:44
RT: 0.00 - 17.01
NL: 1.11E4
m/z= 564.3768-564.3824 F: FTMS + p ESI Full ms [400.00-610.00] MS PC_m_plasma_KO_1167_Fr1a_GPd5_Fr1b_GPd0_120809_07
Chemical Formula: C34H50N3O4+
Exact Mass: 564.3796
5.52
6.37
2.18 2.66 3.92 4.11 5.32 7.35 7.98 9.04 11.25 11.81 12.56 13.69
NL: 1.61E4
m/z= 569.4082-569.4138 F: FTMS + p ESI Full ms [400.00-610.00] MS pc_m_plasma_wt_1178_fr1a_gpd0_fr1b_gpd5_120809_07
Chemical Formula: C34H45D5N3O4+
Exact Mass: 569.4110
5.49
6.32
2.18 4.29 4.72 5.31 8.55 9.35 9.37 9.40 10.99 11.54
Relative Abundance
Time (min)

S8b

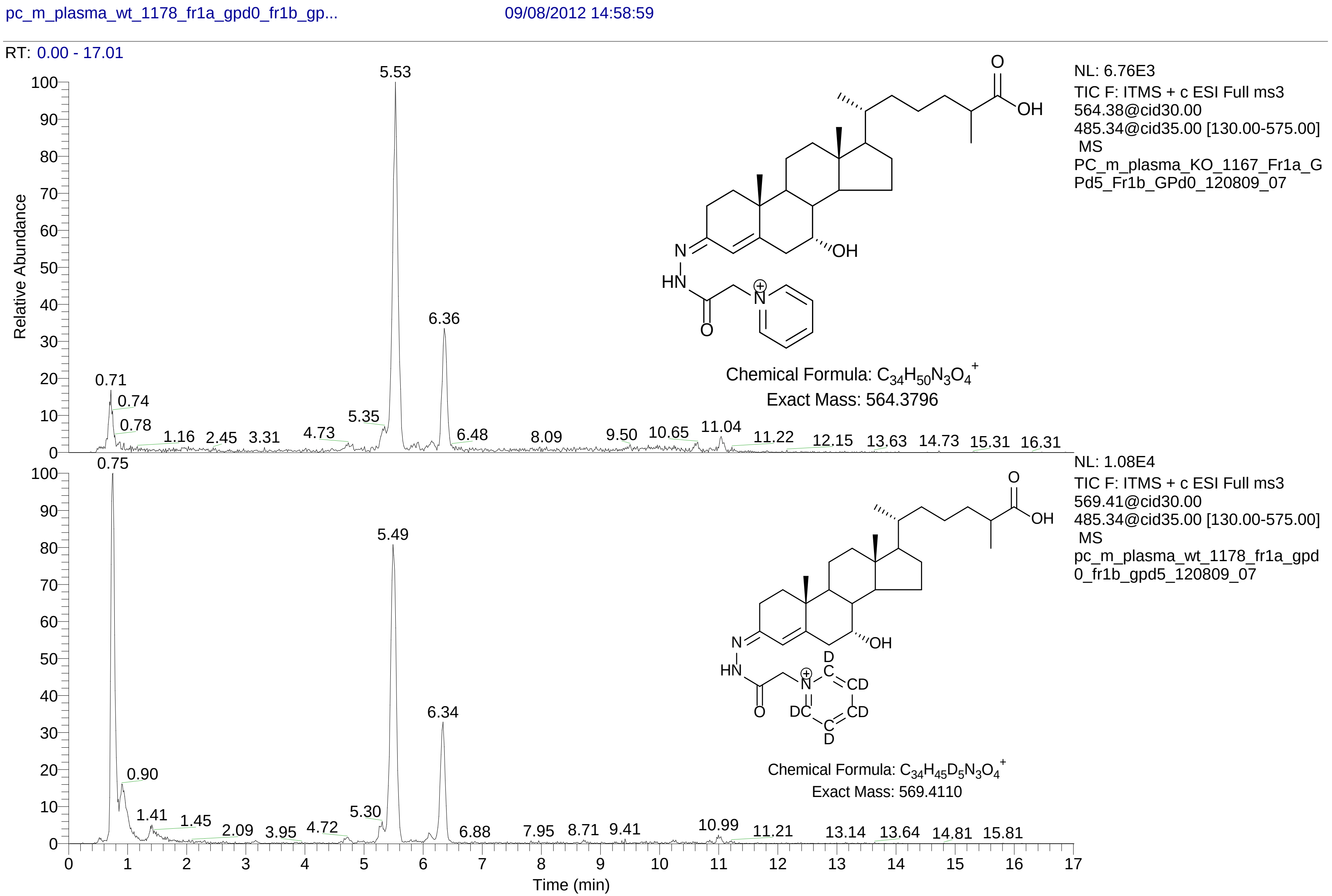
pc_m_plasma_wt_1178_fr1a_gpd0_fr1b_gp...
09/08/2012 14:58:59
RT: 0.00 - 17.01
NL: 6.76E3
TIC F: ITMS + c ESI Full ms3 564.38@cid30.00 485.34@cid35.00 [130.00-575.00] MS
PC_m_plasma_KO_1167_Fr1a_GPd5_Fr1b_GPd0_120809_07
Chemical Formula: C34H50N3O4+
Exact Mass: 564.3796
NL: 1.08E4
TIC F: ITMS + c ESI Full ms3 569.41@cid30.00 485.34@cid35.00 [130.00-575.00] MS
pc_m_plasma_wt_1178_fr1a_gpd0_fr1b_gpd5_120809_07
Chemical Formula: C34H45D5N3O4+
Exact Mass: 569.4110
Relative Abundance
Time (min)
5.53
6.36
0.71
0.74
0.78
5.35
0.75
5.49
6.34
0.90
5.30

S8c

pc_m_plasma_wt_1178_fr1a_gpd0_fr1b_gp... 09/08/2012 14:58:59

pc_m_plasma_wt_1178_fr1a_gpd0_fr1b_gpd5_120809_07 #1110 RT: 5.49 AV: 1 NL: 2.69E3
F: ITMS + c ESI Full ms3 569.41@cid30.00 485.34@cid35.00 [130.00-575.00]

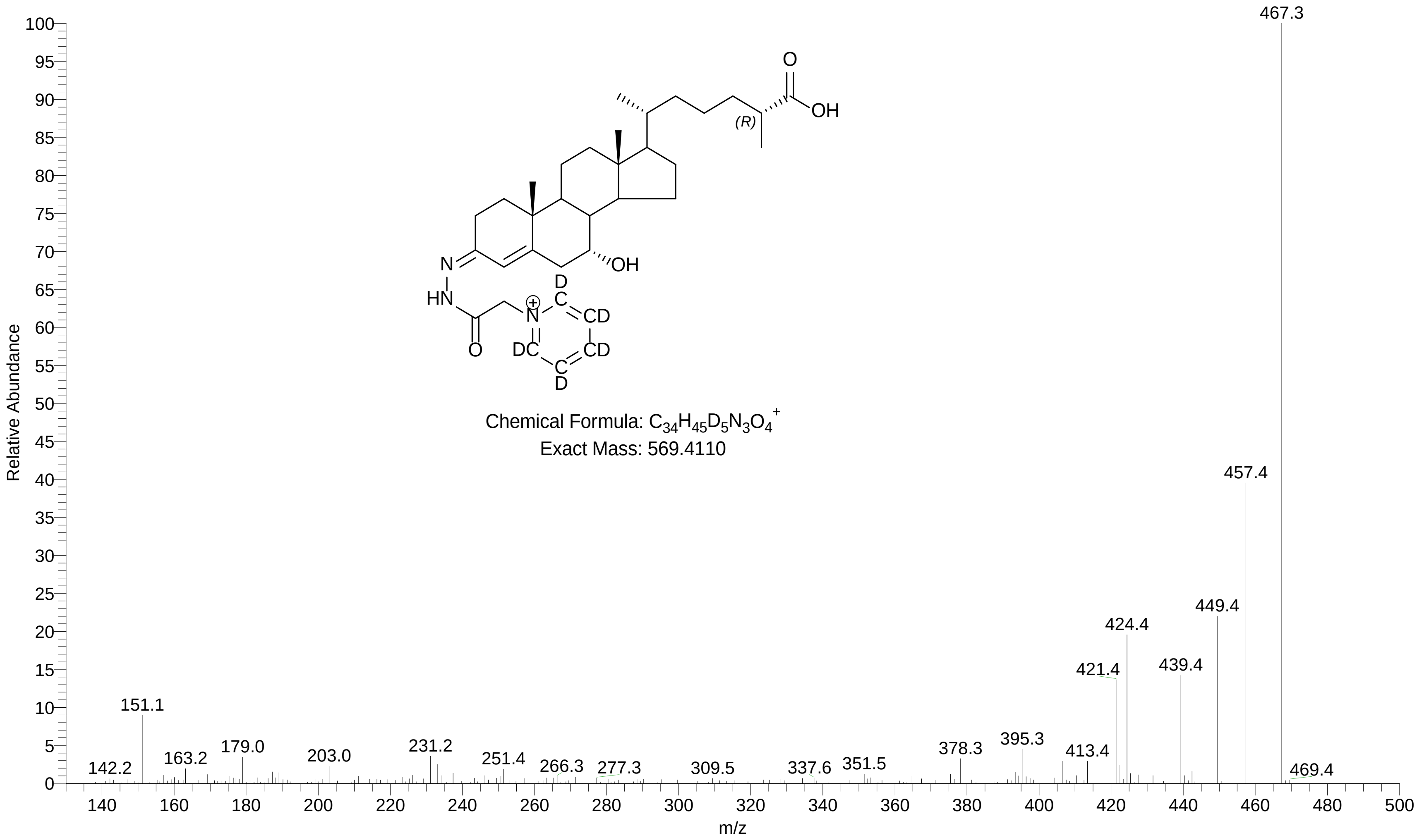

S8d

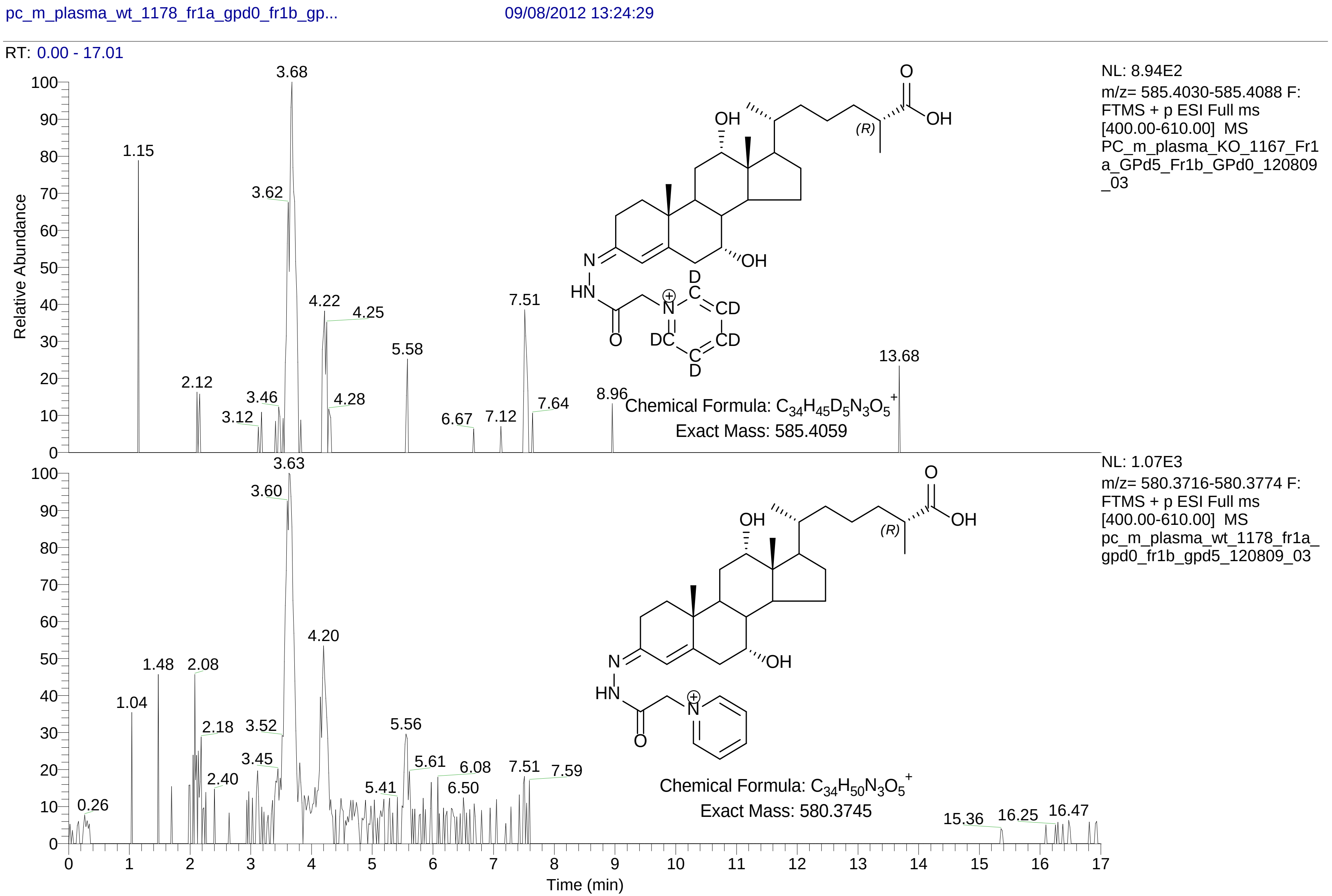
pc_m_plasma_wt_1178_fr1a_gpd0_fr1b_gp...
09/08/2012 13:24:29
RT: 0.00 - 17.01
NL: 8.94E2
m/z= 585.4030-585.4088 F: FTMS + p ESI Full ms [400.00-610.00] MS PC_m_plasma_KO_1167_Fr1a_GPd5_Fr1b_GPd0_120809_03
Chemical Formula: C34H45D5N3O5+
Exact Mass: 585.4059
NL: 1.07E3
m/z= 580.3716-580.3774 F: FTMS + p ESI Full ms [400.00-610.00] MS pc_m_plasma_wt_1178_fr1a_gpd0_fr1b_gpd5_120809_03
Chemical Formula: C34H50N3O5+
Exact Mass: 580.3745
Relative Abundance
Time (min)

S8e

pc_m_plasma_wt_1178_fr1a_gpd0_fr1b_gp... 09/08/2012 13:24:29

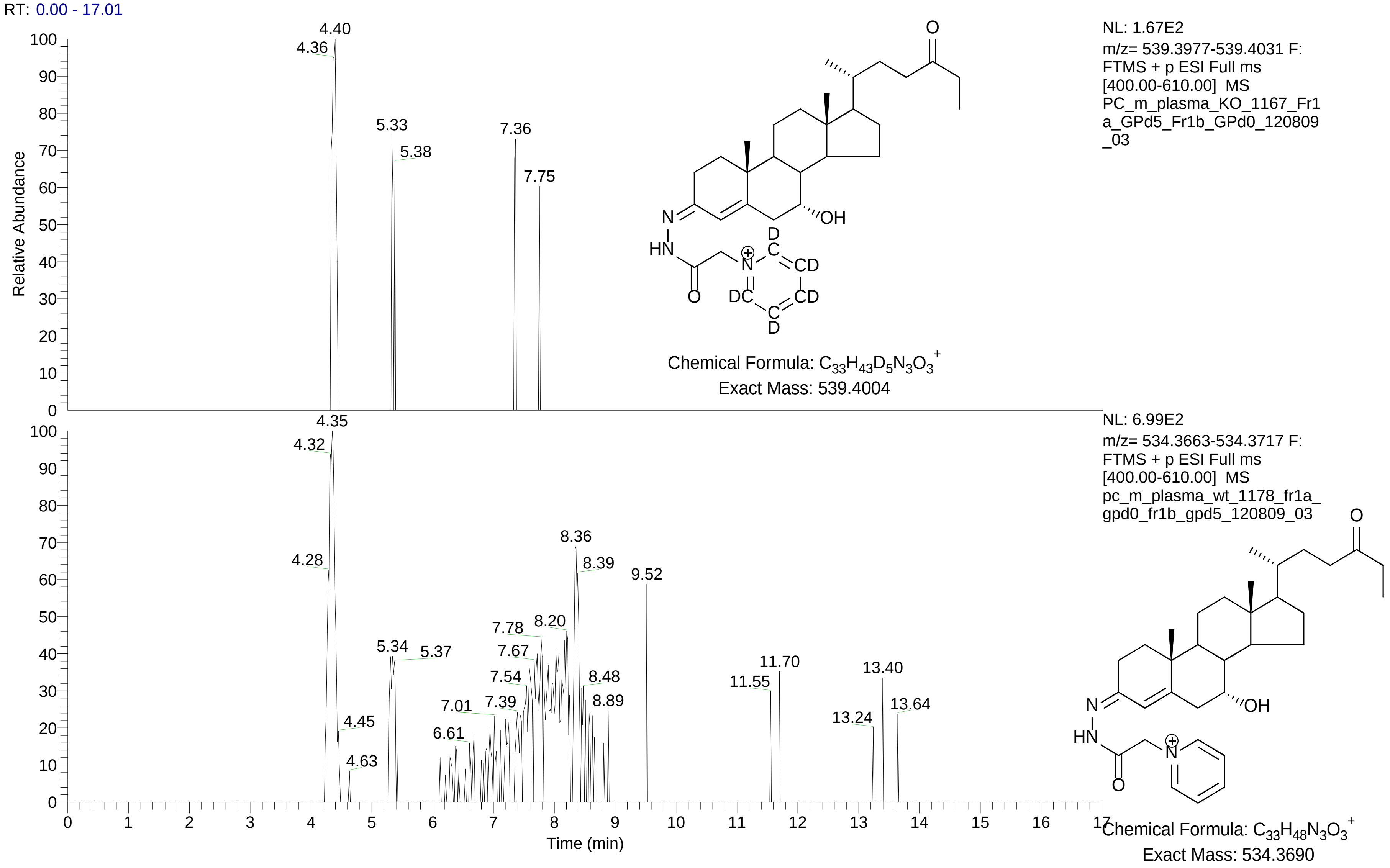

S8f

pc_m_plasma_wt_1178_fr1a_gpd0_fr1b_gp... 09/08/2012 13:24:29

pc_m_plasma_wt_1178_fr1a_gpd0_fr1b_gpd5_120809_03 #884 RT: 4.31 AV: 1 NL: 1.48E2
F: ITMS + c ESI Full ms3 534.41@cid30.00 455.36@cid35.00 [125.00-545.00]

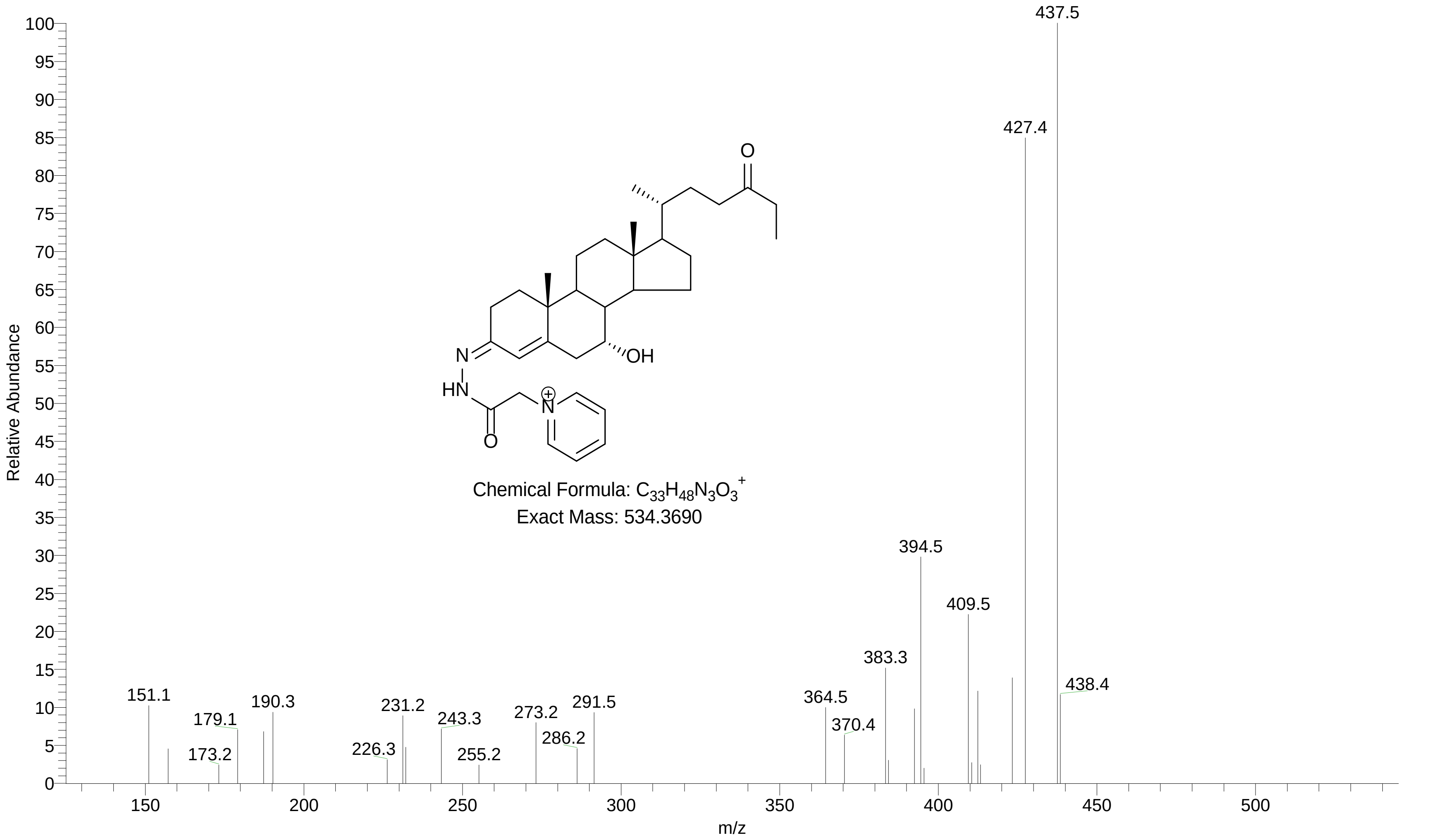

Supplement: Supplementary file 1 [file biomolecules-09-00149-s001.zip › 190327_Biomolecules_Supplementary_Figures_S1_S8_V12.pdf]
